# Remarkable adsorptive removal of nitrogen-containing compounds from hydrotreated fuel by molecularly imprinted poly-2-(1*H*-imidazol-2-yl)-4-phenol nanofibers†

**DOI:** 10.1039/c8ra00543e

**Published:** 2018-02-20

**Authors:** M. S. Abdul-quadir, E. E. Ferg, Z. R. Tshentu, A. S. Ogunlaja

**Affiliations:** Department of Chemistry, Nelson Mandela University P.O. Box 77000 Port Elizabeth 6031 South Africa adeniyi.ogunlaja@mandela.ac.za +27 46 504 3061

## Abstract

Molecularly imprinted polymer (MIP) nanofibers were prepared by the electrospinning of poly 2-(1*H*-imidazol-2-yl)-4-phenol (PIMH) in the presence of various nitrogen containing compounds (N-compounds). Molecularly imprinted polymer nanofibers show selectivity for various target model nitrogen-containing compounds with adsorption capacities of 11.7 ± 0.9 mg g^−1^, 11.9 ± 0.8 mg g^−1^ and 11.3 ± 1.1 mg g^−1^ for quinoline, pyrimidine and carbazole, respectively. Molecular modelling based upon density functional theory (DFT) indicated that hydrogen bond interactions may take place between the lone-pair nitrogen atom of model compounds (quinoline and pyrimidine) and the –OH and –NH groups of the PIMH nanofibers. The adsorption mode followed the Freundlich (multi-layered) adsorption isotherm, which indicated possible nitrogen–nitrogen compound interactions. Molecularly imprinted polymer nanofibers show potential for the removal of nitrogen-containing compounds in fuel.

## Abstract 2

Molecularly imprinted poly-2-(1*H*-imidazol-2-yl)-4-phenol nanofibers fabricated *via* electrospinning displayed excellent selectivity adsorption capacities nitrogen containing compounds (N-compounds) in hydro-treated fuels.

## Graphical abstract

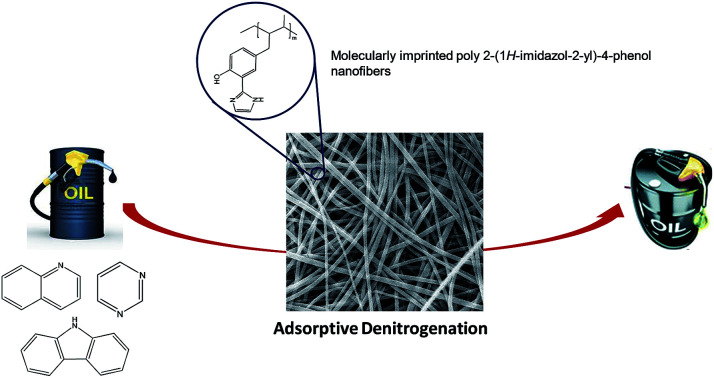

## Introduction

Fuel oil is the major source of energy for industrial machines and most vehicles in many countries of the world, and it is obtained mainly from fossil sources.^1^ The hydrocarbons are mixed with variable quantities of sulfur-, nitrogen-, and oxygen-containing compounds. Refinery feedstocks in general are extremely complicated chemical mixtures in which each heteroatom is present in the form of literally hundreds of different compounds. In general, the presence of organonitrogen compounds in fuel results in fuel instability during storage, due to the formation of gums, colours and sediments, compounds, such as quinoline and related molecules are capable of binding strongly on catalyst surfaces, hence, deactivating refining catalysts.^2–7^ The most harmful effect of nitrogen compounds in fuel is the emission of NOx into the environment which is a major source of pollution that causes many environmental hazards like acid rain, greenhouse effect and photochemical smog.

New severe environmental regulations call for much lower levels of heteroatom-derived pollutants, organosulfur compounds and organosulfur compounds and this has stimulated an intensification of research and development efforts toward new or improved processes.^8^ Due to the harm posed by these gases, the US EPA aimed at improving air quality mandated the reduction of nitrogen content in diesel fuel to ultra-low levels of <1 mg L^−1^,^9^ South Africa is also driving toward achieving the ultra-low levels.

Hydrodenitrogenation (HDN) is currently being employed to eliminate organonitrogen compounds in fuels. These extensively used technologies enabled oil refineries throughout the world for many years to systematically reach a ceiling of *ca.* 70% nitrogen removal, corresponding to a final content of about 0.5 wt% N at typical operating conditions (25–50 atm, 330–350 °C).^10–13^ This indicated that the HDN technique is limited in eliminating organonitrogen compounds, thus requires alternative/complementary techniques such as extraction denitrogenation (EDN), oxidation denitrogenation (ODN), adsorptive denitrogenation (ADN), and biodenitrogenation are currently under study.

Extractive denitrogenation or liquid–liquid extraction is another method that has been applied in the removal of organonitrogen compounds in fuel.^14^ This method suffers from the fact that other compounds can also be extracted along with the organonitrogen compounds. Oxidative denitrogenation (ODN), an alternative/complementary technique to the HDN, involves the oxidation of nitrogen-containing compounds to form oxygenated derivatives. Ishihara *et al.*^15^ reported that nitrogen-containing compounds give N-oxide and other products due to cycle cleavage as well as polymer formation due to the presence of radicals. Ding *et al.*,^16^ recently reported the mild oxidation of pyrimidine to N-oxides with H_2_O_2_ by vanadium-substituted polyoxometalate. However, the reaction did not proceed to the removal of the N-oxides. Recently, our research group^17^ described a technological method for the removal of nitrogen compounds from fuels, *via* oxidation and extractive adsorption. Oxidative denitrogenation is an expensive technique which requires the development of additional plant in the refining industry.

Adsorptive denitrogenation (ADN) is about the most recent method that is being applied to remove organonitrogen compounds in fuel oil. So far, many adsorbents such as activated carbon, zeolites, silica, ion-exchange resins have been used for the ADN,^18–21^ with less selectivity in the removal of organonitrogen compounds in fuels. However, sorbent materials such as molecularly imprinted polymers (MIPs) fabricated through imprinting of templates unto polymers suffice as potential adsorbents for the adsorption of these compounds.^22^ Molecular imprinting of polymers is a technique employed for the introduction of recognition sites into polymeric matrices *via* the formation of bonds between the imprinting molecule (template) and functional groups within the polymer network.^22^ Hence, the need to develop smart polymer nanofiber-based adsorbents [molecularly imprinted polymers (MIPs)] with large surface area-to-volume ratio for the selective removal of sulfonated compounds in fuels.^22^

In this study, we describe the selective adsorption of organonitrogen compounds (quinoline, carbazole and pyrimidine) over molecularly imprinted poly 2-(1*H*-imidazol-2-yl)-phenol (PIMH) nanofibers for the first time by employing the solid phase extraction technique. A combined experimental and computational study was adopted to gain a fundamental understanding of the possible interactions responsible for adsorption. Complete diesel fuel analysis prior and after adsorption was achieved through the use of LECO Pegasus GC × GC-HRT.

## Experimental

### Materials

4-Bromo-2-hydroxybenzaldehyde, 2,2-azo-bis(isobutyronitrile) (AIBN), tetrahydrofuran (THF), nitrogen heterocyclic compounds-quinoline, carbazole and pyrimidine were purchased from Sigma Aldrich, Germany. Acetonitrile (HPLC grade) and methanol (HPLC grade) were purchased from Merck, South Africa.

### Synthesis protocol for molecularly imprinted poly 2-(1*H*-imidazol-2-yl)-4-phenol

The compound, poly 2-(1*H*-imidazol-2-yl)-4-phenol, was synthesized in a similar method reported by Sellergren *et al.*,^23^ with some modifications. This is presented in the ESI, Section A.†

### Polymerization of 2-(1*H*-imidazol-2-yl)-4-vinyl phenol

**Scheme 1:**
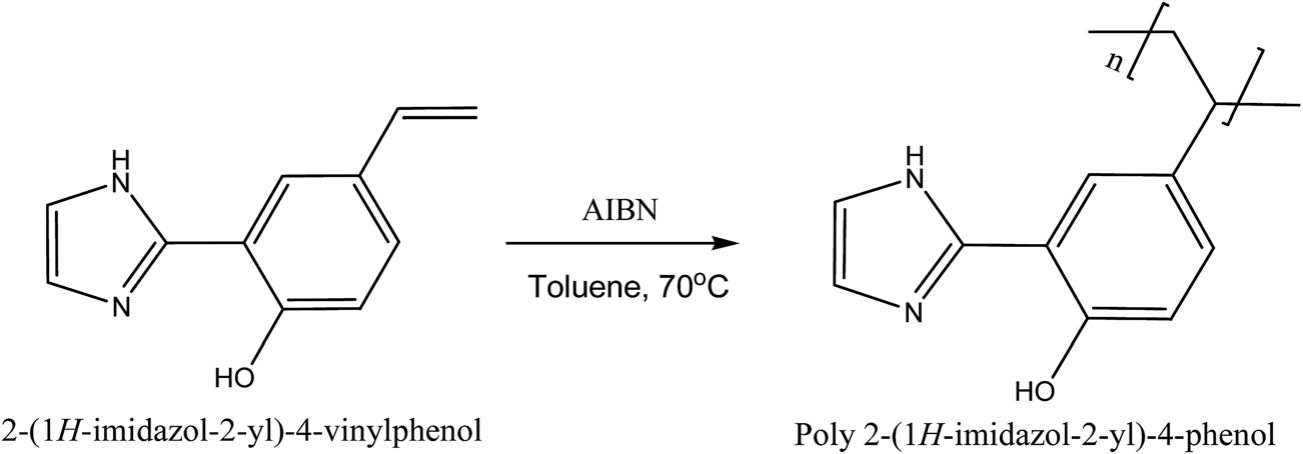
Synthesis of poly 2-(1*H*-imidazol-2-yl)-4-phenol.

2 g of 2-(1*H*-imidazol-2-yl)-4-vinyl phenol, 0.02 g of AIBN and 2 mL of toluene were stirred in 10 mL vial. The mixture was polymerized under argon at 70 °C for 8 h as shown in synthesis Scheme 1. The resulting product, poly 2-(1*H*-imidazol-2-yl)-4-phenol, was dissolved in THF and precipitated out with methanol, filtered and washed with methanol.

### Fabrication of molecularly imprinted poly 2-(1*H*-imidazol-2-yl)-4-phenol (PIMH) nanofibers

Imprinted and non-imprinted poly 2-(1*H*-imidazol-2-yl)-4-vinyl phenol nanofibers were fabricated *via* electrospinning of poly 2-(1*H*-imidazol-2-yl)-4-phenol solutions. Polymer solution for electrospinning was prepared by dissolving 2.2 g of poly 2-(1*H*-imidazol-2-yl)-4-phenol in 4 mL of THF : DMF (1 : 1). The mixture was gently stirred overnight at room temperature to obtain a homogeneous solution for electrospinning. For molecularly imprinted nanofibers, 1 mL (10 mM in acetonitrile) of the respective nitrogen containing compounds containing 20 μL triton X-114 (surfactant agent) was added drop-wise to the dissolved poly 2-(1*H*-imidazol-2-yl)-4-phenol solution. The mixture was further stirred for 2 h to achieve the desirable solution homogeneity for electrospinning (nitrogen containing compounds were not employed in the fabrication of non-imprinted nanofibers).

Polymer solutions were respectively poured into a 25 mL syringe attached to a needle connected to the positive electrode of a high voltage power supply (Series EL, Glassman high voltage Inc). A syringe pump (Model NE-1010, New Era Pump Systems Inc. USA) was used to supply a constant flow of polymer solution from the syringe during the electrospinning process. A voltage of 20 kV was applied to the polymer solution which was pumped at a flow-rate of 0.3 mL h^−1^, with a distance between the needle tip and aluminium collector plate placed at 15 cm. The repulsive electrical forces between charged nanofibers enable them to spread smoothly and the solvent evaporates resulting in solidification while traveling toward the grounded collector. The template molecules (nitrogen containing compounds) imprinted within the nanofibers were removed by washing the nanofibers *via* Soxhlet extraction with a solution mixture of warm-to-hot methanol and acetonitrile (1 : 1) until no templates was detected on the GC.

### Adsorption studies of model nitrogen containing compounds

Nitrogen compounds adsorption studies were performed under batch conditions by weighing a known mass of molecularly imprinted poly 2-(1*H*-imidazol-2-yl)-4-phenol nanofibers (50 mg) into screw-capped vials containing 3 mL of nitrogen containing compounds (120 mg L^−1^) alongside other molecules. The screw-capped vials containing nitrogen compounds were left under mechanical agitation 100 rpm for 12 h. Progress in the adsorption of the various nitrogen containing compounds were followed by withdrawing aliquots for measurement after every hour. Adsorption capacity, *q*_e_ (mg g^−1^) was calculated from eqn (1).1
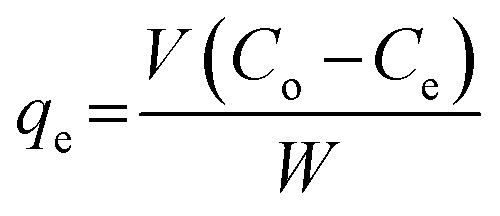
where *C*_o_, *C*_e_, *W* and *V* are the initial concentration (mg L^−1^), equilibrium concentration (mg L^−1^), dry weight of nanofibers (g) and solution volume (L) respectively. Conditioning of adsorbents was carried-out by pre-wetting adsorbents with solvents employed in dissolving the nitrogen compounds.

### Desorption/extraction of absorbed nitrogen containing compounds

Molecularly imprinted poly 2-(1*H*-imidazol-2-yl)-4-phenol nanofibers employed for adsorption were regenerated for re-usability by washing nanofibers with warm solvent mixture of acetonitrile and methanol (1 : 1).

### Solid phase extraction

#### Column preparation

One millilitre plastic syringes were used as column in the SPE manifold. A small amount of glass wool was placed at the bottom of each syringe to prevent loss of the molecularly imprinted nanofibers during sample loading followed by introducing 50 mg of molecularly imprinted nanofibers were loaded into the column. It was conditioned successively with hexane. After each use, the nanofibers in the column was washed with large volumes of dichloromethane and methanol (∼10 mL); and then stored for the next experiment. A typical solid phase extraction (SPE) manifold employed for the study is presented in Fig. S1.†

### Pre-concentration

The molecularly imprinted nanofibers column method was tested with model solutions prior to the selective adsorption and determination of N-compounds in fuel. For N-compound adsorption, 3 mL solution containing known amount of N-compounds in model fuel was employed. The column was preconditioned by passing hexane solution under gravity. After passing of this solution ending, the column was loaded with N-compounds solution. The adsorbed N-compounds on the column were eluted with 5–10 mL portion of methanol : acetonitrile (1 : 1). The eluent was analysed for the determinations of the various N-compound concentrations by GC-FID and GC × GC-HRT.

### Van't Hoff plot

The Van 't Hoff plot was used to determine the thermodynamic parameters such as enthalpy, entropy and Gibbs free energy of adsorption process.^24^ Van 't Hoff experiments for all model organonitrogen compounds were carried out at 30, 35, 40 and 45 °C by using batch adsorption process. Aliquots of analyte solutions are withdrawn at every 20 min interval and analysed by using a GC-FID. Thus, Δ*H*° can be determined from the slope of the linear Van't Hoff plot *i.e.* in *K*_ad_*versus* (1/*T*).^24^ Isothermal Titration Calorimetry (ITC) studies was also conducted and presented in the ESI (Section B).† Molecular interactions between poly 2-(1*H*-imidazol-2-yl)-4-phenol (PIMH) and the various nitrogen containing compounds (quinoline, carbazole and pyrimidine) were modelled and discussed in the ESI, Section B.†

### Instrumentation

FT-IR spectra (4000–400 cm^−1^) were run on Bruker, Tensor 27 platinum ATR-FTIR spectrometer. The ^1^H NMR spectra of ligands and monomer were recorded on a Bruker 400 MHz spectrometer in DMSO-*d*_6_. Thermogravimetric analyses of imprinted and non-imprinted nanofibers were performed using Perkin-Elmer TGA 7 thermogravimetric analyser (TGA). Typically, the samples were heated at a rate of 10 °C min^−1^ under a constant stream of nitrogen gas. The polymer nanofibers were imaged using a TESCAN Vega TS 5136LM scanning electron microscope (SEM). Before images were taken; the various 2-(1*H*-imidazol-2-yl)-4-phenol nanofibers were gold coated to prevent surface charging and to protect the surface material from thermal damage by the electron beam. Nitrogen adsorption/desorption isotherms were measured at 77 K using a TriStar II 3020 3.02 Analyzer by Micromeritics Instrument Corporation to determine surface area and porosity of the nanofibers. Prior to each measurement, nanofibers were degassed at 60 °C for 24 h. The BET surface area, total pore volume and pore size distribution were calculated from these isotherms. Adsorption studies for model compounds was monitored by employing an Agilent 7890A gas chromatograph fitted with flame ionization detector (GC-FID). The GC conditions for the adsorption analyses was started with an oven temperature of 50 °C ramping to 80 °C for 2 min, and then increased to 300 °C at a rate of 20 °C min^−1^, and finally held for 1 min.

LECO Pegasus GC × GC-HRT was employed to monitor the adsorption of organonitrogen compounds in diesel; injection : split injection (100 : 1) at 250 °C; primary column: Stabilwax (Restek), 30 m × 250 μm (0.25 μm); secondary column: Rxi-5 (Restek), 1.5 m × 100 μm (0.1 μm); carrier gas: helium, 1.2 mL per minute constant flow; primary oven program: 40 °C (0.1 minute) to 260 °C (78.4 min) at 3 °C per minute; secondary oven program: 45 °C (0.1 minute) to 265 °C (56 minute) at 3 °C per minute; modulator offset: 15 °C; modulation frequency: 8 seconds; hot time: 2 seconds; MS: LECO Pegasus 4D GC × GC-HRT; ionization: electron ionization at 70 eV; source temperature: 250 °C; stored mass range: 30 to 500 u; acquisition rate: 100 spectra per second.

## Results and discussion

### FT-IR analysis

The FT-IR spectra of non-imprinted nanofibers, carbazole-imprinted nanofibers, pyrimidine-imprinted nanofibers and quinoline imprinted nanofibers are all presented in Fig. S2 of ESI data.† Characteristic absorption bands such as hydroxyl (–OH), imine (–C=N), amine (–NH), (–C=N–C–) and (C=C) groups were observed for all nanofibers. FT-IR analyses of the imprinted compounds are as follows: carbazole-imprinted nanofibers FT-IR (cm^−1^): 3293 *ν*(O–H), 1650 (–NH) bend, 1632 *ν*(C=N), 1626 ν(C=N–C), 1507 *ν*(C=C); pyrimidine-imprinted nanofibers FT-IR (cm^−1^): 3223 *ν*(O-H), 1650 (–NH) bend, 1623 *ν*(C=N), 1631 *ν*(C=N–C), 1503 *ν*(C=C); quinoline-imprinted nanofibers FT-IR (cm^−1^): 3228 *ν*(O–H), 1630 (–NH) bend, 1632 *ν*(C=N), 1623 *ν*(C=N–C), 1506 *ν*(C=C).

### Thermogravimetric analysis (TGA) of non-imprinted and imprinted nanofibers

A weight loss of loosely-bound solvents within the nanofibers were observed at temperatures below 120 °C. All nanofibers presented a single-step decomposition pattern which occur between 375–450 °C.

#### Non-imprinted nanofibers (NIP)

**Fig. 1:**
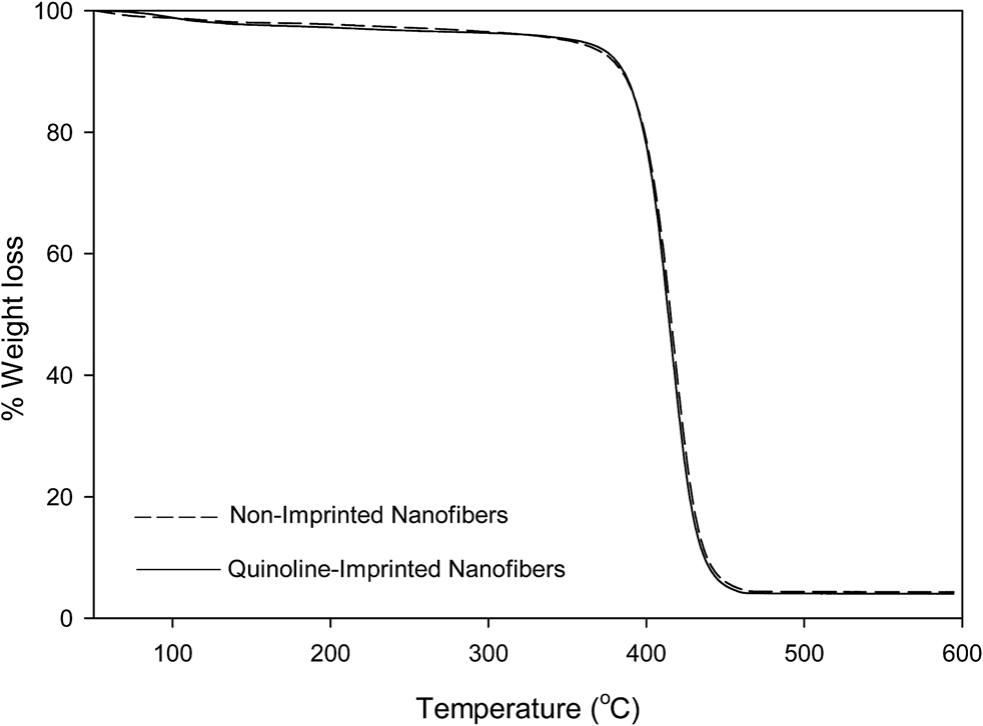
TGA profile of non-imprinted nanofibers and quinoline-imprinted nanofibers (QUNMIP).

**Fig. 2:**
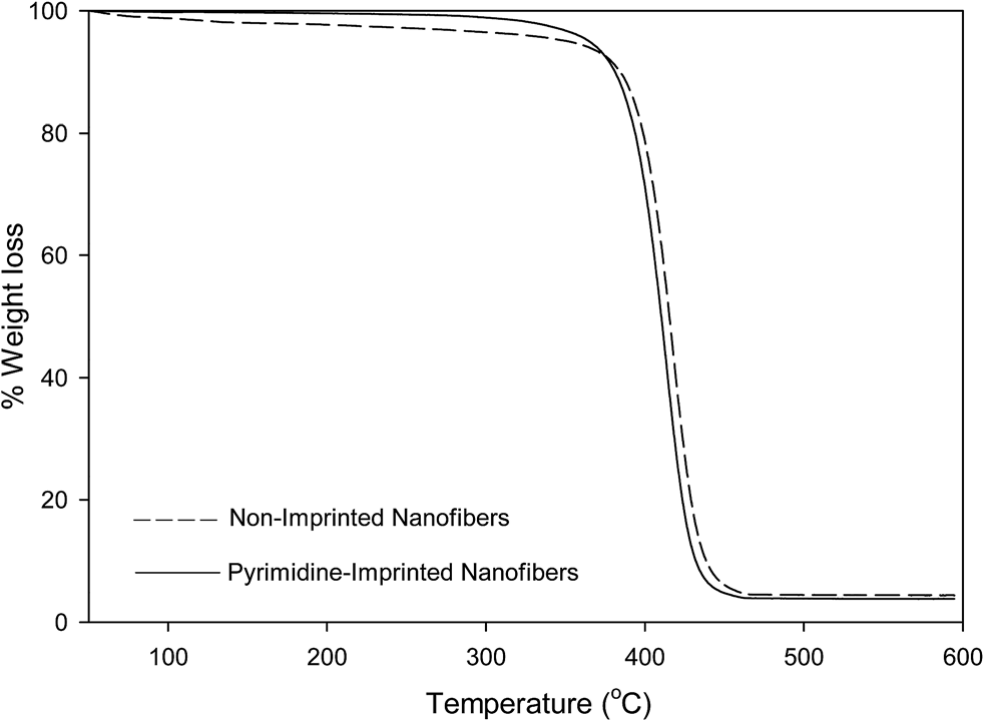
TGA profile of non-imprinted nanofibers and pyrimidine-imprinted nanofibers (PYMMIP).

**Fig. 3:**
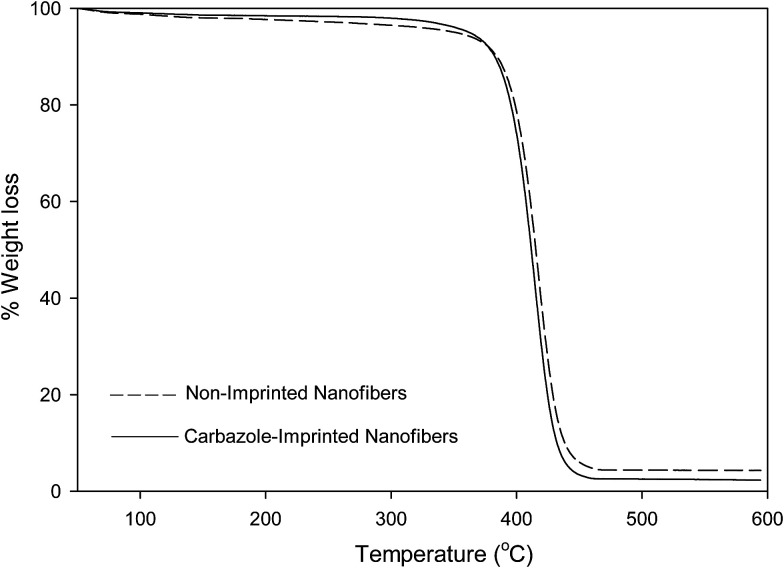
TGA profile of non-imprinted nanofibers and carbazole-imprinted nanofibers (CARMIP).

NIP nanofibers presented a single-step decomposition pattern which occur between 395–440 °C. An observed 3% weight loss around 120 °C was assigned to loosely-bound (intermolecular) solvent within the nanofibers. The decomposition of non-imprinted nanofibers backbone only began to occur at temperatures of around 370 °C to about 440 °C, hence resulting in a total polymer weight loss of 92%, after which the carbon residue remained. The TGA profile of NIP was presented alongside the profiles reported for quinoline-imprinted nanofibers (QUNMIP), pyrimidine-imprinted nanofibers (PYMMIP) and carbazole-imprinted nanofibers (CARMIP) for a better comparison (Fig. 1–3).

#### Quinoline-imprinted nanofibers (QUNMIP)

The quinoline-imprinted nanofibers gave similar decomposition patterns as likened with NIP nanofibers (one step decomposition: 386–440 °C). A 4% weight loss observed at around 110 °C was attributed to loosely-held (intermolecular) solvent molecules within the nanofibers. Followed by a rapid decomposition of quinoline-imprinted polymer backbone at 370 °C to a temperature of about 450 °C (Fig. 1). A total weight loss of 95% was observed.

#### Pyrimidine-imprinted nanofibers (PYMMIP)

The pyrimidine-imprinted nanofibers (PYMMIP) nanofibers presented a distinct weight loss between 380 and 430 °C. Pyrimidine-imprinted nanofibers displayed high stability to around 300 °C, this confirmed that the nanofibers were dry and free of solvents. Gradual breakdown of polymer backbone only began to occur at temperatures between 390 and 430 °C, with a total weight loss of 96% (Fig. 2).

#### Carbazole-imprinted nanofibers (CARMIP)

The carbazole-imprinted nanofibers (CARMIP) presented one distinct weight losses at around 390 °C and 430 °C. A 2% weight loss observed at around 110 °C was attributed to loosely-bound solvent within the nanofibers. Followed by a rapid decomposition of quinoline-imprinted polymer backbone at 370 °C to a temperature of about 450 °C (Fig. 3). A total weight loss of 94% was observed.

The thermal stabilities of molecularly imprinted nanofibers obtained *via* TGA analysis are in the order of; pyrimidine-imprinted nanofiber > carbazole-imprinted nanofibers > quinoline imprinted nanofibers.

### Scanning electron micrograph (SEM) and energy dispersive spectroscopy (EDS) of non-imprinted and imprinted nanofibers

**Fig. 4:**
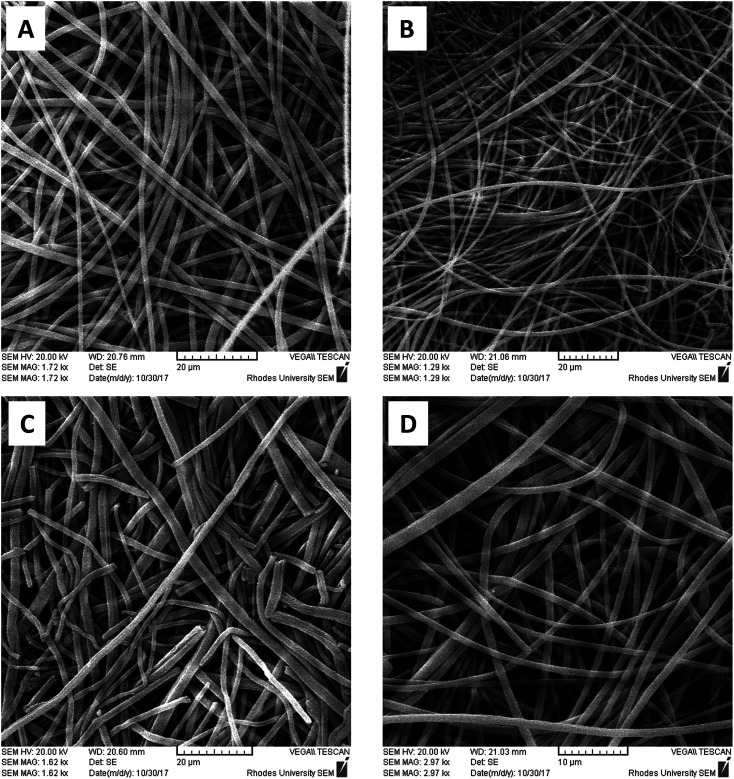
Scanning electron micrograph (SEM) images of (A) non-imprinted nanofibers, (B) quinoline-imprinted nanofibers (QUNMIP), (C) pyrimidine-imprinted nanofibers (PYMMIP) and (D) carbazole-imprinted nanofibers (CARMIP).

The SEM micrographs of non-imprinted and imprinted nanofibers after washing and drying are presented in Fig. 4A–D. A diameter range of between 100–270 nm was observed for all nanofibers (Fig. 4A–D). SEM images also indicated a break in the fiber strand of pyrimidine-imprinted nanofibers (PYMMIP), this may probably be due to the imprinting effect.

Energy Dispersive Spectroscopy (EDS) images for the various non-imprinted and imprinted nanofibers after washing and drying are presented in Fig. S3.† Chemical characterization of nanofibers after template removal were determined, it is worth noting that EDS presents qualitative data and therefore cannot be employed as quantitative data. EDS study confirmed that the chemical integrity of the nanofibers was preserved even after washing the templates off the nanofibers.

### BET surface area

A Brunauer–Emmett–Teller (BET) model was used to calculate the specific surface area and a Barrett–Joyner–Halenda (BJH) model was used to calculate the pore volume distribution and the average pore size of (A) non-imprinted nanofibers, (B) quinoline-imprinted nanofibers (QUNMIP), (C) pyrimidine-imprinted nanofibers (PYMMIP) and (D) carbazole-imprinted nanofibers (CARMIP).

A decrease in the surface area of the nanofibers upon imprinting was due to pores being created by organonitrogen compounds as seen in the reported pore sizes (Table 1). The result also displayed changes in nanofibers pore sizes (Å), thus corresponding to the sizes of imprinted compounds. The random fibrous mesh obtained as shown in Fig. 4 may have influenced the pore size values obtained as no visible pores were seen on the SEM images of the nanofibers. Hence, the various pores sizes obtained may be the determined external pores.

**Table 1:** Surface area and pore volumes of non-imprinted and imprinted nanofibers^a^

Adsorbents (nanofibers)	Surface area (m^2^ g^−1^)	Pore size (Å)
NIP	75.34	53.25
PIMMIP	57.91	55.78
QUNMIP	63.14	53.08
CARMIP	49.11	55.54

a Non-imprinted nanofibers (NIP), quinoline-imprinted nanofibers (QUNMIP), pyrimidine-imprinted nanofibers (PIMMIP) and carbazole-imprinted nanofibers (CARMIP).

### Adsorption studies

#### Nitrogen containing compounds selectivity studies

Adsorption assays were carried out to evaluate the loading capacity and selectivity of imprinted poly 2-(1*H*-imidazol-2-yl)-4-phenol (PIMH) nanofibers. 50 mg of the imprinted adsorbents were added to vials and mixed with 3 mL solution mixture of nitrogen containing compounds (quinoline, carbazole and pyrimidine), dibenzothiophene and naphthalene (120 mg L^−1^ each).

The corresponding adsorption assays were first carried out by employing non-imprinted nanofibers followed by using imprinted nanofibers. The suspension was left under mechanical agitation at 150 rpm for 12 h as described in the adsorption studies (Fig. S4†). The use of non-imprinted gave a maximum adsorption of 0.62 mg g^−1^, 0.51 mg g^−1^ and 0.63 mg g^−1^ respectively for quinoline, carbazole and pyrimidine.

Relatively moderate adsorption capacities with no selectivity were observed for non-imprinted when NIP nanofibers were employed. However, high adsorption capacities were observed when molecularly imprinted nanofibers were employed to target specific N-compounds: (i) quinoline-imprinted nanofibers (QUNMIP) presented 11.7 ± 0.9 mg g^−1^, quinoline (Fig. S5†) (ii) pyrimidine-imprinted nanofibers (PYMMIP) presented 11.9 ± 0.8 mg g^−1^, pyrimidine (Fig. S6†) and (iii) carbazole-imprinted nanofibers (CARMIP) presented 11.3 ± 1.1 mg g^−1^, carbazole (Fig. S7†). The molecularly imprinted nanofibers presented a much higher adsorption capacity as compared to the data observed when molecularly imprinted polybenzimidazole (PBI) was employed, an adsorption capacity of 4.8 mg g^−1^ was observed with PBI.^17^

The non-specific binding nature on non-imprinted nanofibers gave rise to the non-selective adsorption reported as compared to the imprinted nanofibers whose binding sites alongside cavities created *via* imprinting allows for selective adsorption.^26^ A reduction of <20 mg L^−1^ observed for dibenzothiophene and naphthalene was attributed to the chemical properties as well as the high surface area presented by the polymer nanofibers. The reduction dibenzothiophene and naphthalene concentrations could have resulted from π–π stacking between absorbent and adsorbates (dibenzothiophene and naphthalene).

Theoretical calculation *via* subtraction of the unabsorbed N-compounds indicated that concentrations of 118.8, 117.3 and 116.4 mg L^−1^ for pyrimidine, quinoline and carbazole, respectively, are required for complete adsorption.

### Imprinting factor (*k*)

The imprinting constant (*k*) is defined as the ratio of the adsorption capacity of imprinted nanofibers (*Q*_MIP_) to the adsorption capacity of the non-imprinted nanofibers (*Q*_NIP_). The higher the value of *k*, the better is the imprinting effect.
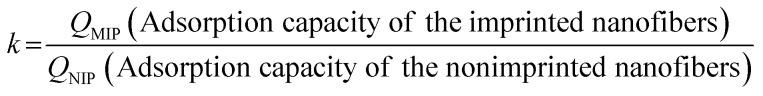
where *k* is the imprinting factor, *Q*_NIP_ (mg g^−1^) is the adsorption capacity of the non-imprinted nanofibers and *Q*_MIP_ (mg g^−1^) is the adsorption capacity of imprinted nanofibers.

The imprinting factor (*k*) values for quinoline-imprinted nanofibers is 18.92, pyrimidine-imprinted nanofibers is 18.56, and carbazole-imprinted nanofibers is 22.16, respectively (Table S1†).

### Adsorption kinetics

Adsorption kinetic studies using molecularly imprinted nanofibers showed that nitrogen molecule adsorption was initially fast due to the availability of surface adsorption and thereafter adsorption rate slowed as surface saturation is reached, thus, limiting further nitrogen molecules penetration (Fig. S4†).

**Fig. 5:**
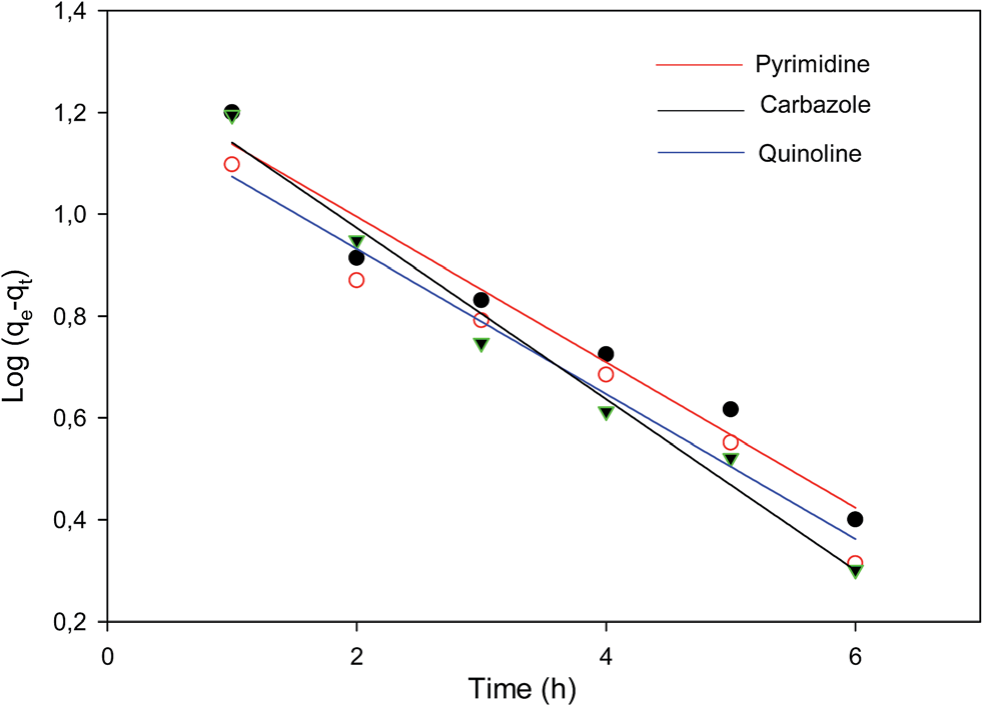
Pseudo-first-order plot of the various nitrogen containing compounds (pyrimidine, carbazole and quinoline).

The kinetic mechanism that controls the adsorption process under batch study was monitored by using pseudo-first-order model (eqn (2)) and pseudo-second-order model (eqn (3)). In pseudo-first-order model the occupation rate of the adsorption sites is proportional to the number of unoccupied sites, while the pseudo-second-order assumes that adsorption takes place *via* a chemical reaction process *i.e.* chemisorption process. Kinetic studies were carried out by using molecularly imprinted nanofibers (50 mg) contained in 3 mL of 120 mg L^−1^ N-compounds solution.2
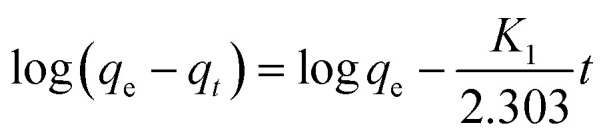
3
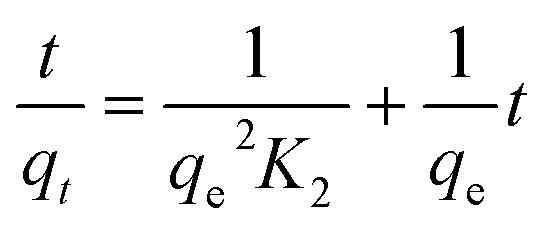
where *q*_e_ and *q*_*t*_ (mg g^−1^) are the amounts of N-compounds adsorbed on the adsorbent at equilibrium and time *t*, respectively. *K*_1_ (h^−1^) is the pseudo-first-order adsorption rate constant and was calculated by plotting the log(*q*_e_ − *q*_*t*_) *versus t* (Fig. 5). The pseudo-first-order and pseudo-second-order parameters (coefficients) are presented in Table 2. Based on the obtained correlation coefficients (*R*^2^), carbazole, quinoline and pyrimidine fitted the pseudo-first-order model. The pseudo-second-order plot is presented in Fig. S8.†

**Table 2:** Kinetic data of pseudo-first-order and pseudo-second-order

	Pseudo-first-order kinetics		Pseudo-second-order kinetics	
	*k* (h^−1^)	*R* ^2^	*k*_2_ (g mg^−1^ h^−1^)	*R* ^2^
Quinoline	0.349	0.9746	0.024	0.8020
Carbazole	0.387	0.9647	0.066	0.7812
Pyrimidine	0.327	0.9617	0.054	0.4849

### Adsorption isotherms

The adsorption behaviour of nitrogen containing compounds on imprinted nanofibers was followed using the Langmuir and Freundlich isothermal equations (eqn (4) and (5)).4
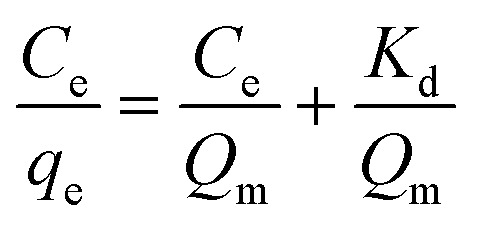
5
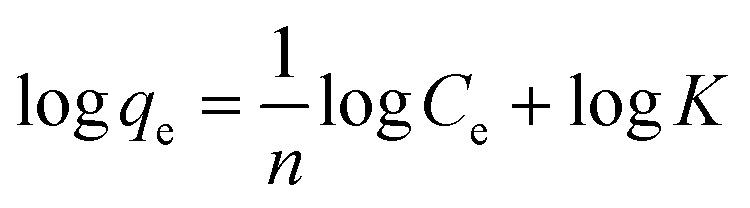
where *q*_e_ (mg g^−1^) and *C*_e_ (mg g^−1^) are the amount adsorbed at equilibrium and the equilibrium concentration, (*Q*_m_), is the theoretical maximum adsorption capacity at monolayer (mg g^−1^), and *K*_d_ is the Langmuir constant (related to the affinity of adsorption sites).^26^ Freundlich constants *k* and *n* indicating adsorption capacity and intensity respectively were determined from the linear plot of log *q*_e_ against log *C*_e_. Freundlich isotherm indicated a multiple layered adsorption on adsorbents, which may possibly infer nitrogen compound-nitrogen compound interactions on the adsorbent. While Langmuir equation obtained from a plot of *C*_e_/*q*_e_ against *C*_e_ is probably proof of chemical adsorption, which may usually mean monolayer adsorption on the surface of adsorbents.^26^

**Fig. 6:**
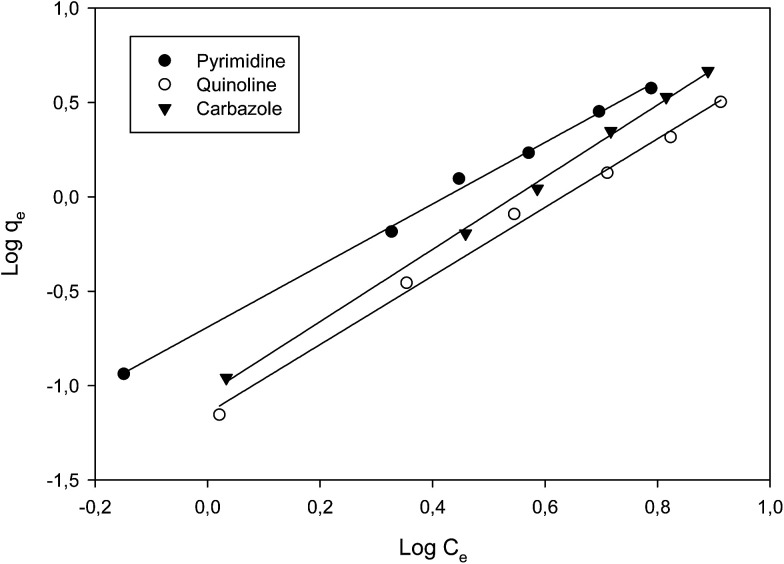
Freundlich plot of the various nitrogen containing compounds (pyrimidine, carbazole and quinoline).

From the plots, Freundlich adsorption equation fitted better (larger correlation coefficient *R*^2^, Fig. 6 and Table 3) with equilibrium data as compared to the Langmuir parameters and we also observed that Freundlich constant ‘*n*’ value lies within the range 1 to 10, thus indicating that adsorption on the nanofibers are favourable.^26^ The Langmuir parameters plot is presented in Fig. S9.†

**Table 3:** Parameters of Langmuir adsorption model and Freundlich adsorption model

	Langmuir parameters		Freundlich parameters	
	*Q*_m_	*R* ^2^	*n*	*R* ^2^
Quinoline	10.7	0.9134	1.45	0.9945
Carbazole	8.9	0.9567	1.71	0.9983
Pyrimidine	11.2	0.9127	1.02	0.9969

### Thermodynamic studies

#### Van't Hoff plot

**Fig. 7:**
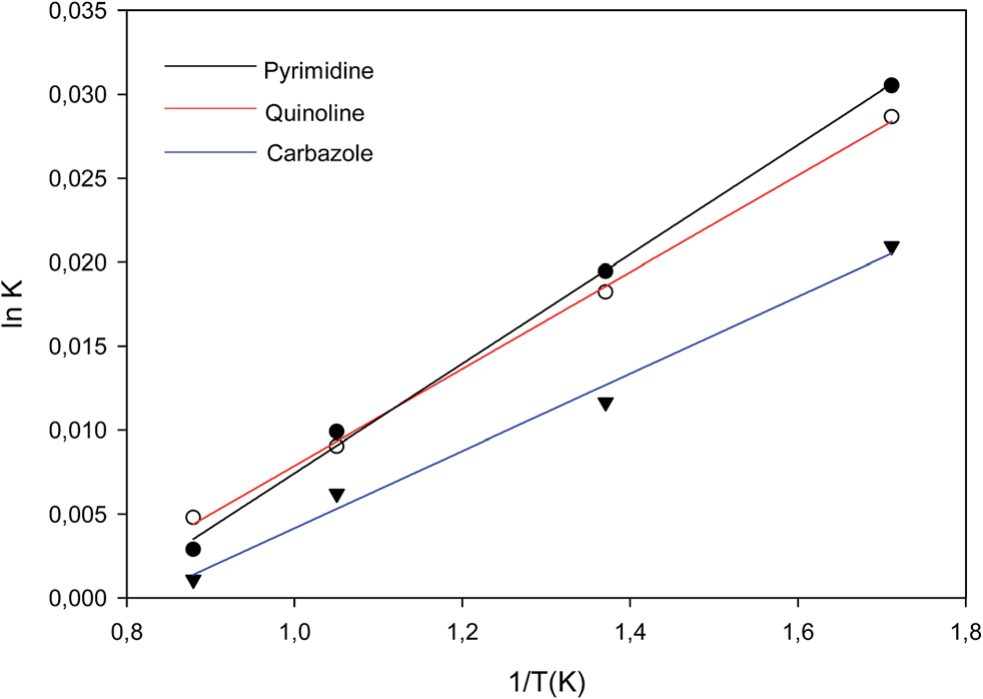
Van't Hoff Plot of adsorption equilibrium constant *K*_ad_ for adsorption of nitrogen containing compounds onto imprinted nanofibers.

Fig. 7 shows the Van't Hoff's plot for carbazole, quinoline and pyrimidine adsorptions and the calculated thermodynamic values (Δ*H*°, Δ*S*° and 
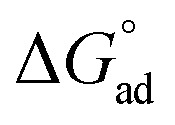
) for N-compounds are shown in Table 4. Negative 
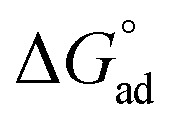
 observed on all interactions indicated feasibility and spontaneity of the adsorption processes.^25^ The negative Δ*H*° values observed for all nitrogen containing compounds pyrimidine, carbazole and quinoline confirmed the exothermic nature of the overall-sorption process. The positive value of Δ*S*° observed for all nitrogen containing compounds suggests increased randomness at the solid/solution interface with some structural changes (in the adsorbate and adsorbent) as well as the binding affinity of the various nitrogen containing compounds to the imprinted nanofibers.

**Table 4:** Thermodynamic properties obtained from Van't Hoff plot^a^

	Free energy (Δ*G*_b_) (kJ mol^−1^)	Enthalpy (Δ*H*_b_) (kJ mol^−1^)	Entropy (Δ*S*_b_) (kJ mol^−1^)
Quinoline	−62.43	−0.2710	0.2086
Pyrimidine	−52.09	−0.2396	0.1740
Carbazole	−11.10	−0.1911	0.0366

a Temperature = 298 K.

The result obtained from Van't Hoff plot is in agreement with the data obtained through DFT studies and isothermal titration calorimetry (ITC) (please see Section B of the ESI†). Briefly, DFT studies revealed that hydrogen bond interactions may take place between the lone-pair nitrogen atom of N-compounds (quinoline and pyrimidine) and the –OH, –NH groups of the PIMH nanofibers. Carbazole on the other hand offered weak interactions (π–π interactions). Thermodynamic parameters obtained from isothermal titration calorimetry (ITC) revealed that quinoline/PIMH and pyrimidine/PIMH interactions are exothermic, while carbazole/PIMH is endothermic.

### Continuous flow adsorption studies

Continuous flow adsorption technique was employed for the adsorption of pyrimidine, quinoline and carbazole compounds. Breakthrough volumes were evaluated, as they represent the evolution of the concentration of a solution as a function of parameters such as contact time between liquid and solid phase, solvent concentration and temperature. 50 mg of molecularly imprinted nanofibers were packed into a cylindrical tube attached to the tip of a syringe containing 5 mL of respective N-compounds. The molecularly imprinted nanofibers were easily contained in the tube without leaving much space and the packing was tightened by conditioning the material with solvent at 0.5 mL h^−1^. Adsorption progressed as respective N-compounds flow through the conditioned sorbent.

**Fig. 8:**
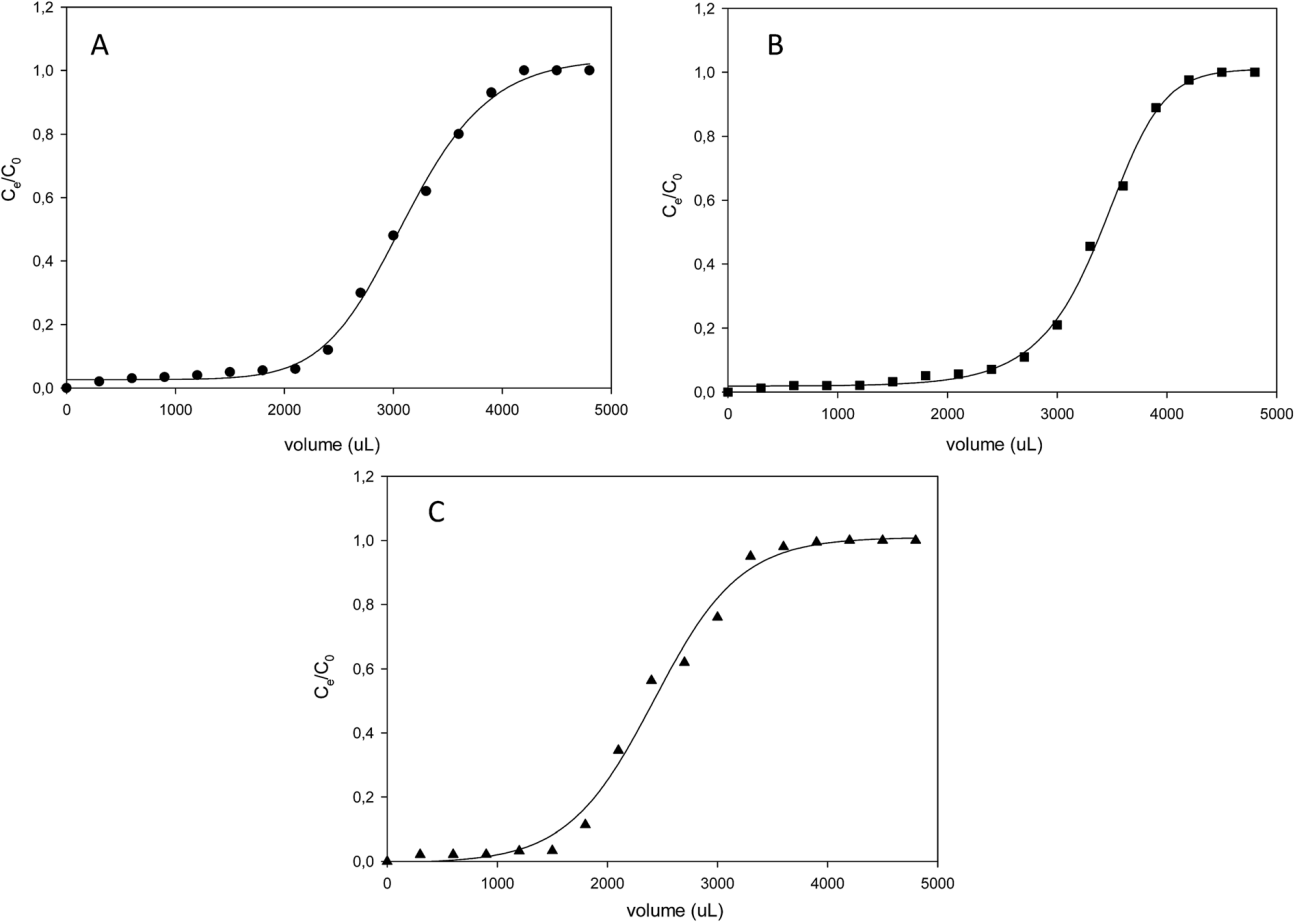
Breakthrough curves of the various organonitrogen compounds (A) quinoline (B) pyrimidine and (C) carbazole while using their various molecularly imprinted nanofibers.

From the adsorption curve, the maximum amount of nitrogen containing compounds retained falls within the range of 0.9–1.5 mL (900–1500 μL). The breakthrough curves of the N-compounds solutions obtained are presented in Fig. 8. *C*_o_ was the initial concentration (g L^−1^) of the nitrogen compounds and *C*_e_ was the eluted concentration of the nitrogen compounds. The linear capacity of the column (*n*_s_), the capacity factor of the solute (*k*) and percentage recovery (*r*) are calculated from eqn (6)–(8).^22,26^ and presented in Table 5.6
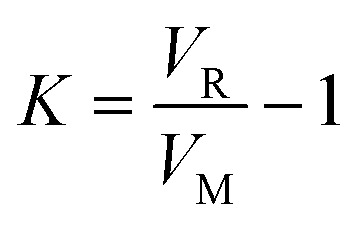

7 *n*_s_ = *V*_M_*KC*_o_

8
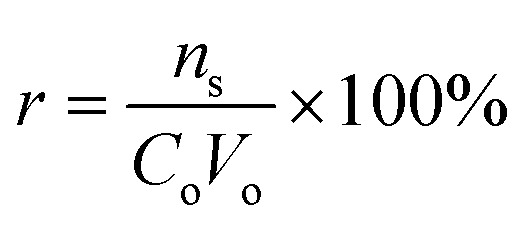
where *V*_o_ is the initial volume of the analyte (nitrogen solutions), breakthrough volume (*V*_B_), retention volume (*V*_R_) and hold-up volume (*V*_M_) of the analyte (nitrogen solutions).

**Table 5:** Influence of nitrogen containing compounds upon adsorption on molecularly imprinted nanofibers

N-compounds	*V*_B_ (mL)	*V*_R_ (mL)	*V*_M_ (mL)	*K*	*n*_s_ × 10^−4^	*r*%
Pyrimidine	2.1	3.2	0.3	9.7	3.8	63.3
Quinoline	2.3	3.0	0.2	14.0	3.4	56.7
Carbazole	1.6	2.4	0.3	7.0	2.5	41.7

A recovery rate in the order of pyrimidine > quinoline > carbazole was observed for the continuous flow study.

### Adsorbents reusability studies

Reusability studies on the imprinted nanofibers were carried out using solid phase extraction (SPE) technique.^27^ Solid phase extraction (SPE) is an increasingly useful sample preparation technique. The rebinding adsorption capacities of imprinted nanofibers decreased significantly for all nitrogen compounds as we move from 1st adsorption cycle to the 3rd cycle for carbazole (Fig. S10†), quinoline (Fig. S11†) and pyrimidine (Fig. S12†). This reduction in adsorption capacities upon employing the nanofibers for several cycles indicated that the imprinting integrity may have been compromised.^26^Table 6 presented the various concentrations absorbed after each cycle. GC-FID chromatograms of model fuel before and after adsorption with molecularly imprinted nanofiber is presented in Fig. S13.†

**Table 6:** Various amounts of nitrogen containing compounds unabsorbed after each cycle^a^

	First adsorption cycle (mg L^−1^)	Second adsorption cycle (mg L^−1^)	Third adsorption cycle (mg L^−1^)
Carbazole	116.4 ± 2.9	113.2 ± 2.9	112.7 ± 3.2
Quinoline	117.3 ± 2.8	115.9 ± 3.9	115.2 ± 3.4
Pyrimidine	118.8 ± 2.9	115.6 ± 3.2	115.2 ± 3.9

a NB: Initial concentration = 120 mg L^−1^.

### Adsorption of nitrogen compounds in diesel fuel

**Fig. 9:**
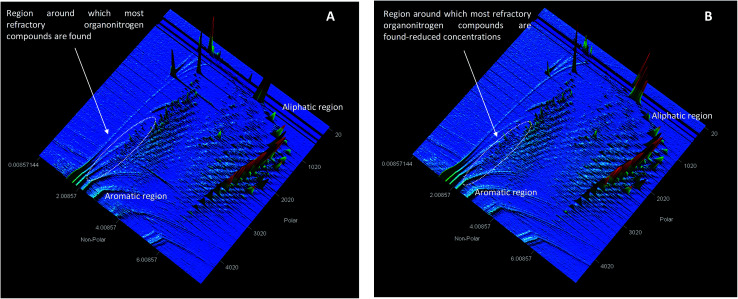
GC × GC-high-resolution TOF-MS surface plot showing (A) surface contour plot of hydrotreated diesel (XIC) and (B) surface contour plot (XIC) after adsorption of hydrotreated diesel fuel.

Hydro-treated diesel was denitrogenated by using molecularly imprinted 2-(1*H*-imidazol-2-yl)-4-phenol polymer nanofibers on solid phase extraction (SPE). Prior to denitrogenation, alkylated organonitrogen compounds in diesel were identified by employing LECO Pegasus 4D GC × GC-HRT (Fig. 9).

Adsorption studies for the removal of organonitrogen compounds was carried out by weighing 150 mg of imprinted nanofibers (comprising of 50 mg quinoline-imprinted, 50 mg carbazole-imprinted and 50 mg pyrimidine-imprinted nanofibers) as adsorbent. Adsorption proceeded under SPE manifold by conditioning imprinted nanofibers at a vacuum pressure of 20 in Hg with a solution of hexane followed by loading the hydrotreated diesel (1 mL hydrotreated diesel in 2 mL of hexane). Cyclohexane was employed to wash interfering molecules from the sorbent and finally N-compounds were eluted by using a mixture of acetonitrile : methanol (1 : 1).

GC × GC-high-resolution TOF-MS surface plot confirmed a reduction in the presence of alkylated organonitrogen compounds in the hydrotreated fuel (Fig. 9). According to the MS data some of the identified alkylated organonitrogen compounds peak area reduced after adsorption studies (Table 7).

**Table 7:** Some alkylated organonitrogen compounds found before and after the adsorption of hydrotreated fuel

Name	Formula	R.T. (s)	Base Mass	Area (Before adsorption)	Area (After adsorption)
Cumidine	C_9_H_13_N	1723.39, 3.35143	120.0932	4676	ND
1-Phenyl-1-propanamine	C_9_H_13_N	1915.33, 3.32572	106.0777	95 933	ND
4-Pentyloxyaniline	C_11_H_17_NO	2930.96, 5.05715	109.1012	47 285	ND
1-Methyl-2,5-dipropyldecahydroquinoline	C_16_H_31_N	4002.58, 2.34857	194.1088	7818	ND
3-(*N*,*N*-Dimethylamino)-9-methylcarbazole	C_15_H_16_N_2_	4002.58, 2.48982	224.1195	42 756	1002
9*H*-Carbazol-3-amine, 9-ethyl-	C_14_H_14_N_2_	3954.6, 2.50286	210.1401	30 379	ND
2-Butyl-1-pyrroline	C_8_H_15_N	347.883, 2.91429	83.0855	25 596	ND

As observed in Table 7, imprinted nanofibers show promise owing to its high surface area and chemical characteristics, thus, enabling the adsorption of refractory organonitrogen compounds. Though, some complexities were observed as the adsorbent also wiped out some non-imprinted compounds. The ability of the polymer nanofibers to adsorb these compounds was attributed to possible hydrogen bonding interactions between adsorbent and these compounds, 1-phenyl-1-propanamine and 4-pentyloxyaniline and 2-butyl-1-pyrroline (Table 7), which could easily interact *via* hydrogen bond formation with the polymer nanofibers. Imprinted nanofibers was unable to eliminate 3-(*N*,*N*-dimethylamino)-9-methylcarbazole completely due to the highly alkylated nature of the compound, thus, inhibiting interactions with the active sites of the adsorbent.

Many solid adsorbents have been employed for denitrogenation of fuels and these adsorbents have proven to be worthwhile in terms of general performance. The use of imprinted nanofibers is relatively new and limited literature exists. However, several investigations of ADN using solid sorbents such as MOFs have been reported.^28,29^ They observed that MOFs with high porosity absorbed higher concentrations of nitrogen containing compounds (NCCs).^29–31^ A study reported by Nuzhdin *et al*.^29^ indicated that a total of 1.3 mmol of nitrogen containing compounds (NCCs) was removed by 1 g of MIL-101(Cr). Results obtained with the use of molecularly imprinted nanofibers in this study could be said to be comparable with the results reported by Nuzhdin *et al*.^29^ and some others reported in the literature.^29^ The use of poly 1,1′-binaphthyl-2,2′-diol nanofibers for the adsorption of quinoline and isoquinoline in a model simulated fuel presented an adsorption capacity of 2.2 and 2.4 mg g^−1^, respectively.^32^ These capacities falls short of the value reported in the current study, hence, confirming an improvement in the fabricated material through imprinting process. Therefore, the use of molecularly imprinted nanofibers as adsorbent in the adsorption of NCCs offers improved surface area, porosity (cavities), adsorption capacities and selectivity.^31^

### Theoretical studies

The atomic level interaction of the various nitrogen containing compounds (quinoline, carbazole and pyrimidine) with poly 2-(1*H*-imidazol-2-yl)-4-phenol were predicted by molecular interaction studies using B3LYP functional with a basis set 6-311G++(d,p) using the Gaussian09 software (calculated at 298 K) under tight convergence criteria.^33^ Further details on DFT calculations such as HOMO–LUMO band gap, hardness (*η*), softness (*σ*), electronegativity (*χ*), and chemical potential (*μ*) calculations are provided in ESI (Section B).†

DFT calculations was carried out for the interaction of N-compounds (quinoline, pyrimidine and carbazole) with 2-(1*H*-imidazol-2-yl)-4-phenol to understand the mechanism of adsorption. Studies reported includes HOMO and LUMO energy positions, HOMO–LUMO energy gap (Δ*E*), hardness (*η*), softness (*σ*), electronegativity (*χ*) and chemical potential (*μ*) of the adsorbent and adducts.^34^

#### HOMO–LUMO positions

Molecular orbital energy diagram of 2-(1*H*-imidazol-2-yl)-4-phenol indicated that the HOMO position centers around the –OH and –NH and aromatic rings of the ligand, 2-(1*H*-imidazol-2-yl)-4-phenol, while the LUMO position originates around the imidazoline ring.

**Fig. 10:**
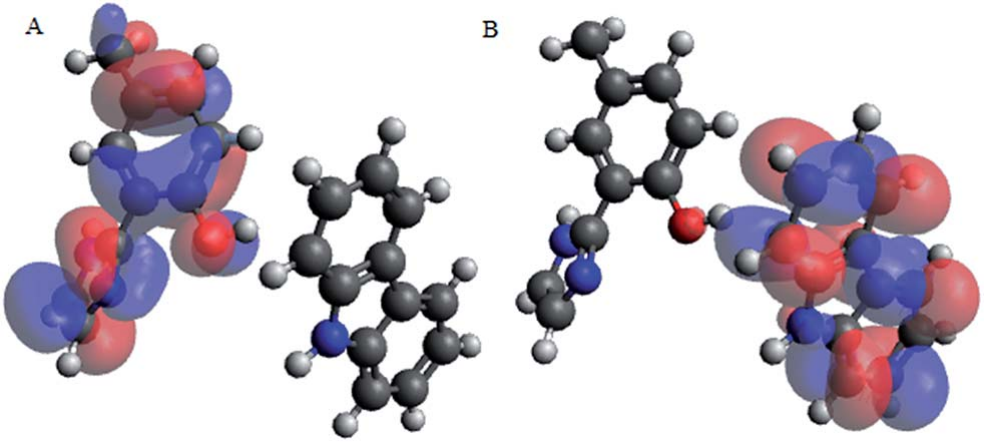
HOMO and LUMO location of adduct between poly 2-(1*H*-imidazol-2-yl)-4-phenol polymer unit and carbazole (PIMH–CAR) (A) HOMO and (B) LUMO. Blue, red and grey colours represent nitrogen, oxygen and carbon atoms respectively.

**Fig. 11:**
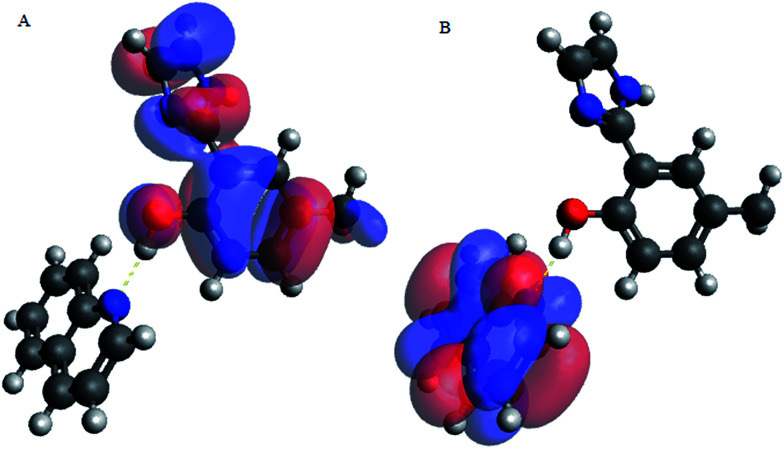
HOMO and LUMO location of adduct between 2-(1*H*-imidazol-2-yl)-4-phenol polymer unit and quinoline (PIMH–QUN), (A) HOMO and (B) LUMO. Blue, red and grey colours represent nitrogen, oxygen and carbon atoms respectively.

**Fig. 12:**
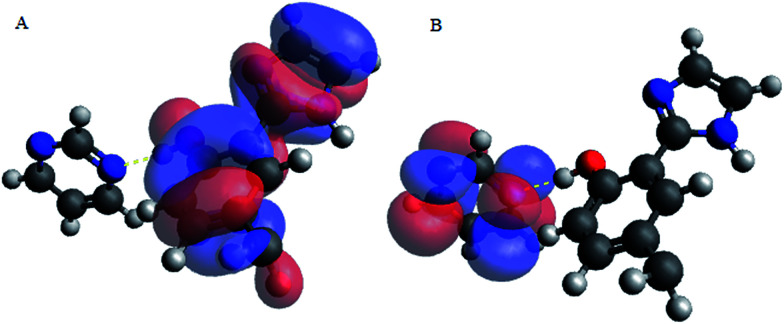
HOMO and LUMO location of adduct between poly 2-(1*H*-imidazol-2-yl)-4-phenol polymer unit and pyrimidine (PIMH–PYM), (A) HOMO and (B) LUMO. Blue, red and grey colours represent nitrogen, oxygen and carbon atoms respectively.

Upon interacting with N-compounds (quinoline, pyrimidine and carbazole), HOMO position of the adducts originates from 2-(1*H*-imidazol-2-yl)-4-phenol, while LUMO centers mostly around each nitrogen containing compounds (Fig. 10–12). This clearly indicated that interactions between the 2-(1*H*-imidazol-2-yl)-4-phenol and N-compounds (quinoline, pyrimidine and carbazole) mainly occur through electron donation from the HOMO to the LUMO (Fig. 10–12). The possible formation of π–π interaction (π–π stacking) between aromatic rings of 2-(1*H*-imidazol-2-yl)-4-phenol and N-compounds as well as hydrogen bonding was observed.

Images showing the HOMO–LUMO positions of 2-(1*H*-imidazol-2-yl)-4-phenol and carbazole, 2-(1*H*-imidazol-2-yl)-4-phenol and quinoline and 2-(1*H*-imidazol-2-yl)-4-phenol and pyrimidine is presented in Fig. 10, 11 and 12, respectively.

#### HOMO–LUMO energy gap

The HOMO–LUMO gap describes the stability and resistance of molecules, and it also predicts activity between species by providing the electrical transport properties as well as electron carrier and mobility in molecules. The HOMO and LUMO energies of 2-(1*H*-imidazol-2-yl)-4-phenol, are −5.35 and 0.49 eV, respectively. High ionization potential is indicated by low HOMO energy (better electron donor), while high electron affinity is indicated by high LUMO energy (better electron acceptor). The energy gap of 2-(1*H*-imidazol-2-yl)-4-phenol which depends on the HOMO and LUMO energies was observed to be 4.88 eV.

Molecular interactions between the various nitrogen compounds with 2-(1*H*-imidazol-2-yl)-4-phenol resulted in a decrease in HOMO–LUMO energy gap when compared to the HOMO–LUMO gap of the nitrogen compounds, thus further indicating interactions. 2-(1*H*-imidazol-2-yl)-4-phenol–carbazole adduct (PIMH–CAR), 2-(1*H*-imidazol-2-yl)-4-phenol–pyrimidine adduct (PIMH–PYM) and 2-(1*H*-imidazol-2-yl)-4-phenol–quinoline adduct (PIMH–QUN) all presented lower HOMO–LUMO gap as compared to the values obtained for carbazole (CAR), pyrimidine (PYM), quinoline (QUN) and 2-(1*H*-imidazol-2-yl)-4-phenol (PIMH) (Table S2†). According to hard and soft acids bases (HSAB), hard acids interact more strongly with hard bases, while soft acids interact more strongly with soft bases.^28^ The order of N-compound hardness and softness is pyrimidine (PYM) < quinoline (QUN) < carbazole (CAR), thus, indicating that pyrimidine is more reactive and carbazole is least reactive. Molecules with large electronegativity can be considered as stronger electron acceptors.^34^ Electronegativity data agrees with the HOMO–LUMO diagram which indicates that electrons are donated by 2-(1*H*-imidazol-2-yl)-4-phenol (PIMH) and accepted N-compounds (quinoline, pyrimidine and carbazole). Some electronic structure identifiers of the studied adducts are presented in Table 8.

**Table 8:** Some electronic structure identifiers of the studied adducts

Compounds	Hardness (*η*)	Softness (*σ*)	Electronegativity (*χ*)	Chemical potential (*μ*)
PIMH	2.94	0.34	2.44	−2.44
Carbazole (CAR)	2.37	0.42	3.31	−3.31
Pyrimidine (PYM)	4.05	0.25	2.89	−2.89
Quinoline (QUN)	2.45	0.41	3.84	−3.84
PIMH–CAR	2.27	0.44	3.16	−3.16
PIMH–PYM	1.84	0.54	3.42	−3.42
PIMH–QUN	1.73	0.58	3.50	−3.50

## Conclusions

Molecular imprinting on 2-(1*H*-imidazol-2-yl)-4-phenol enhanced adsorption capacities and selectivity for individual nitrogen compounds due to their specific binding nature and high adsorption capacities are described. A better regression *R*^2^ presented by Freundlich isotherm confirmed multilayer adsorption attributed to the interactions between imprinted nanofibers and nitrogen compounds, and possibly between nitrogen molecules. The nanofibers displayed excellent adsorption properties when employed under SPE conditions to explore hydrophobic interactions (van der Waals or dispersion forces) and hydrophilic interactions (hydrogen bonding, pi–pi interactions, dipole–dipole interactions). Thermodynamic parameters obtained from isothermal titration calorimetry (ITC) revealed that quinoline–PIMH and pyrimidine–PIMH interactions are exothermic in nature, while carbazole–PIMH is endothermic in nature. Molecular modelling also confirmed that 2-(1*H*-imidazol-2-yl)-4-phenol indicated that hydrogen bond interactions may take place between the lone-pair nitrogen atom of N-compounds (quinoline and pyrimidine) and the –OH, –NH groups of the PIMH nanofibers. Carbazole on the other hand offers weak interactions. Further DFT also confirmed the feasibility of π–π interactions between the imidazole rings and the aromatic N-compounds. The complex nature of N-compounds in fuel complicate the structure/function approach on MIPs for targeting these unwanted compounds.

## Conflicts of interest

There are no conflicts to declare.

## Supplementary Material

RA-008-C8RA00543E-s001
