# Supplementary material for: Remarkable adsorptive removal of nitrogen-containing compounds from hydrotreated fuel by molecularly imprinted poly-2-(1H-imidazol-2-yl)-4-phenol nanofibers
Source: RSC Adv. 2018 Feb 20;8(15):8039–50. doi: 10.1039/c8ra00543e (PMC9078548; doi:10.1039/c8ra00543e)
Supplement: RA-008-C8RA00543E-s001 [file RA-008-C8RA00543E-s001.pdf]

Electronic Supplementary Material (ESI) for RSC Advances.
This journal is © The Royal Society of Chemistry 2018

Remarkable adsorptive removal of nitrogen-containing compounds from hydrotreated fuel by molecularly imprinted poly-2-(1*H*-imidazol-2-yl)-4-phenol nanofibers.

*M. S. Abdul-quadir[a], E. E. Ferg[a], Z. R. Tshentu[a], and A. S. Ogunlaja[a],**

[a]Department of Chemistry, Nelson Mandela University, P.O. Box 77000, Port Elizabeth 6031, South Africa

Supplementary Data

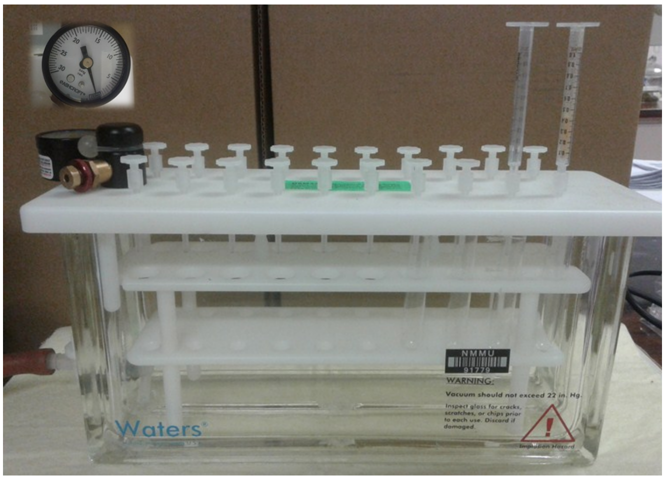

Figure S1**:** A typical Solid Phase Extraction (SPE) Vacuum Manifold.

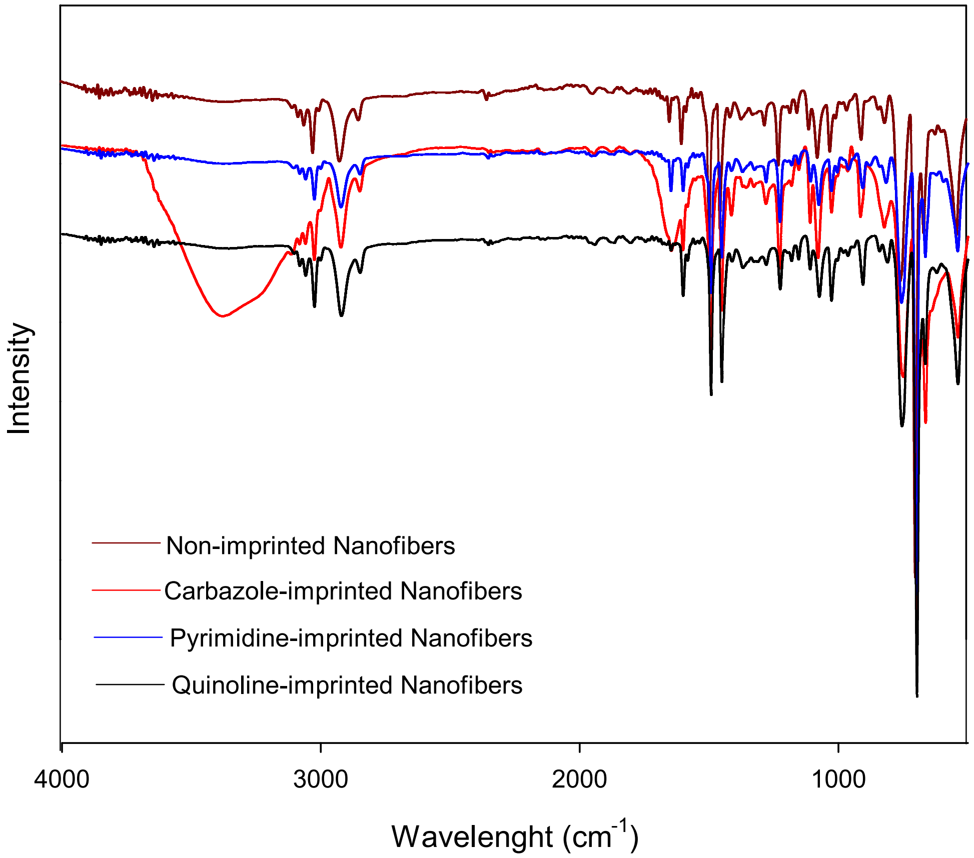

Figure S2: FTIR spectra of (i) non-imprinted nanofibers (ii) carbazole-imprinted nanofibers (iii) pyrimidine-imprinted nanofibers and (iv) quinoline imprinted nanofibers.

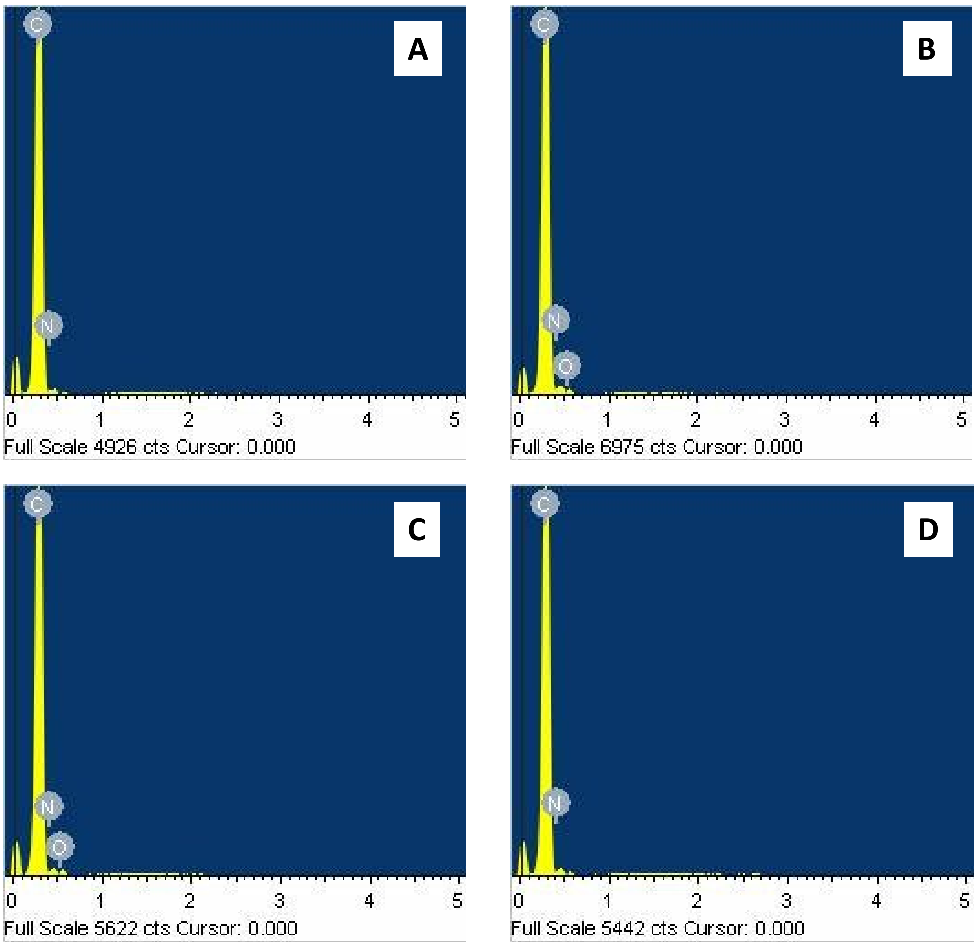

Figure S3 Energy Dispersive Spectroscopy (EDS) of (a) non-imprinted nanofibers, (b) pyrimidine imprinted nanofibers, (c) carbazole imprinted nanofibers, and (d) quinoline imprinted nanofibers.

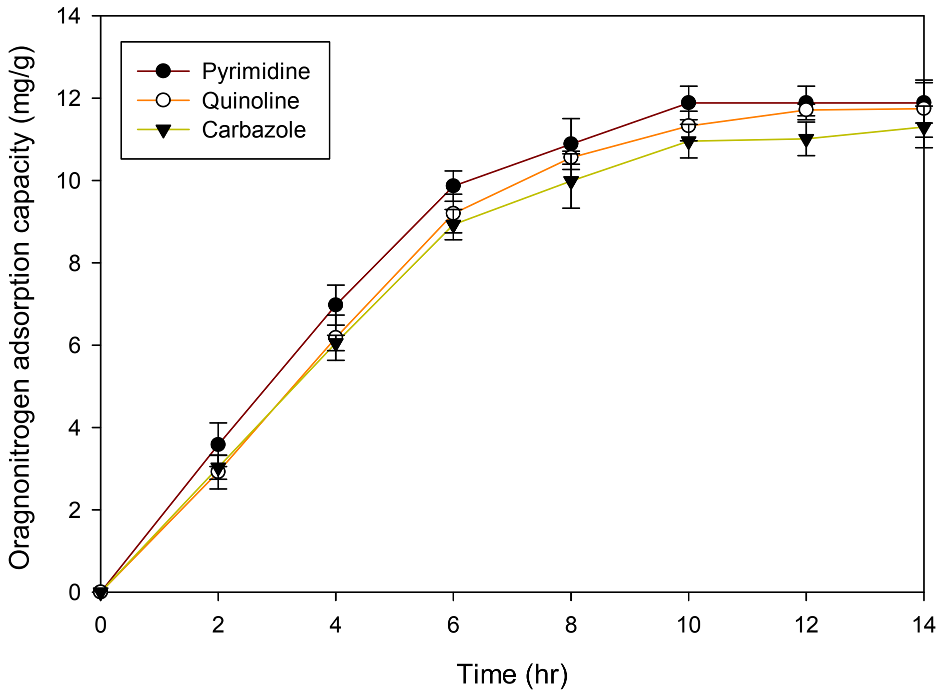

Figure S4. Kinetic of adsorption of the various nitrogen compounds over imprinted nanofibers.

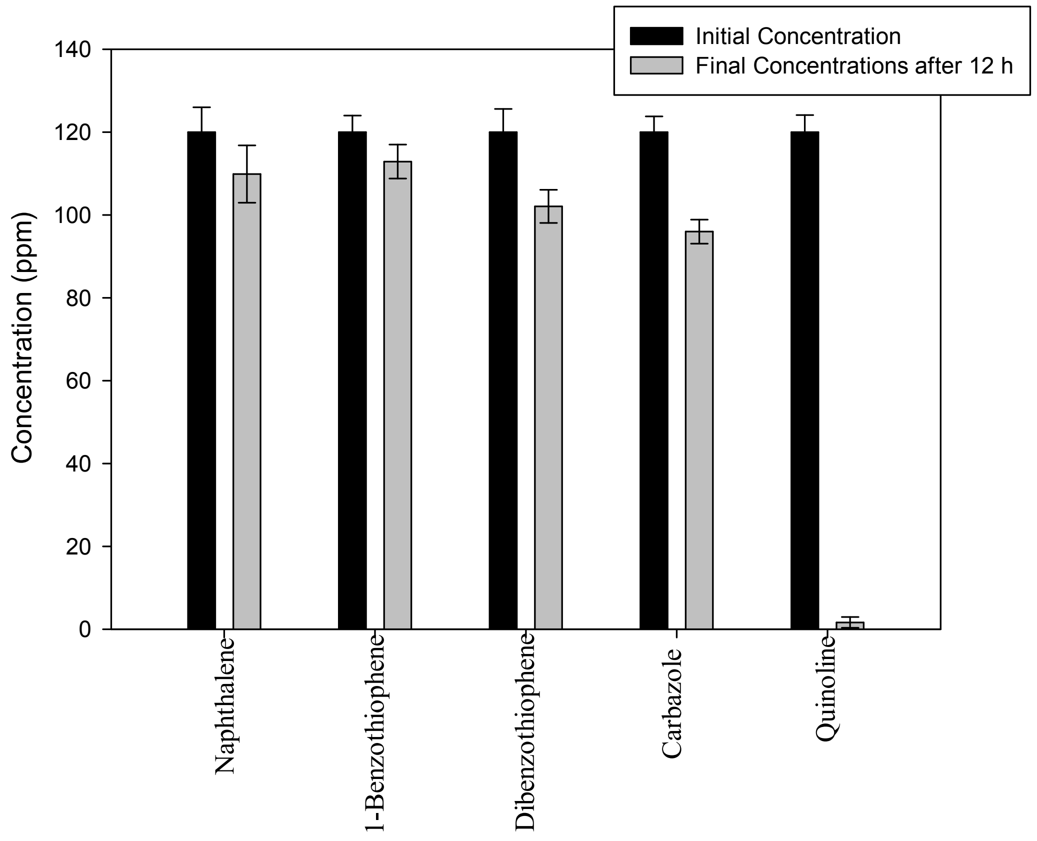

Figure S5. Adsorption selectivity of quinoline using quinoline-imprinted nanofibers.

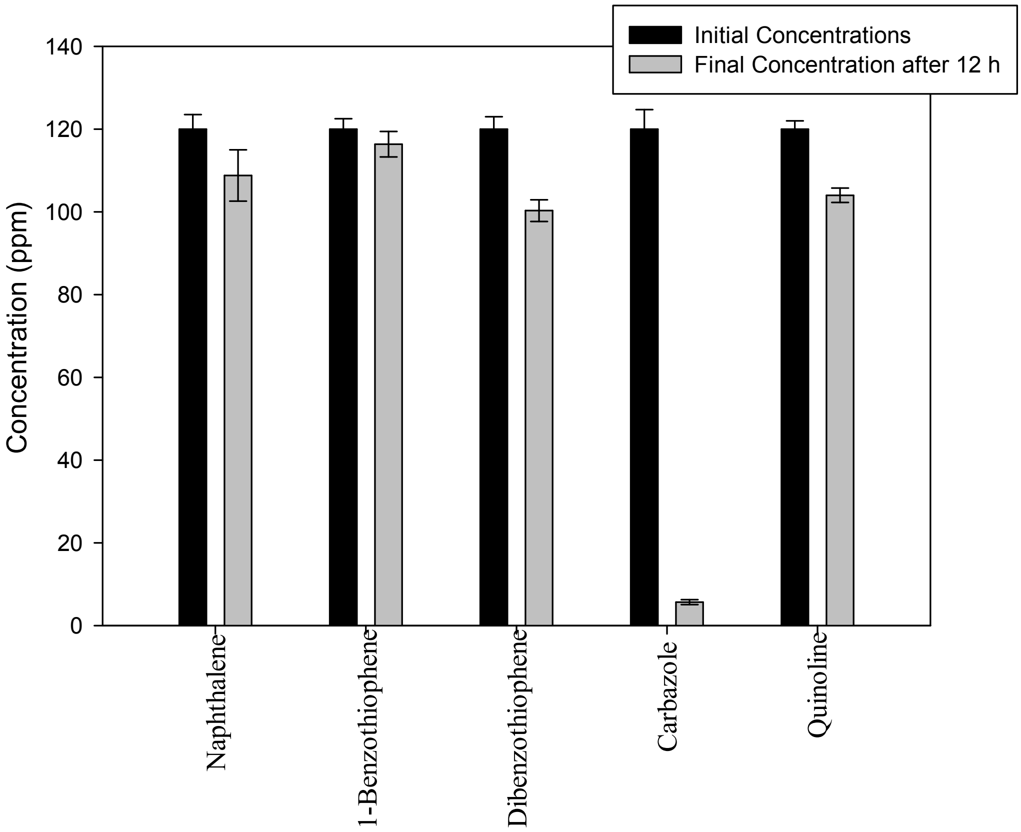

Figure S6. Adsorption selectivity of carbazole using carbazole-imprinted nanofibers.

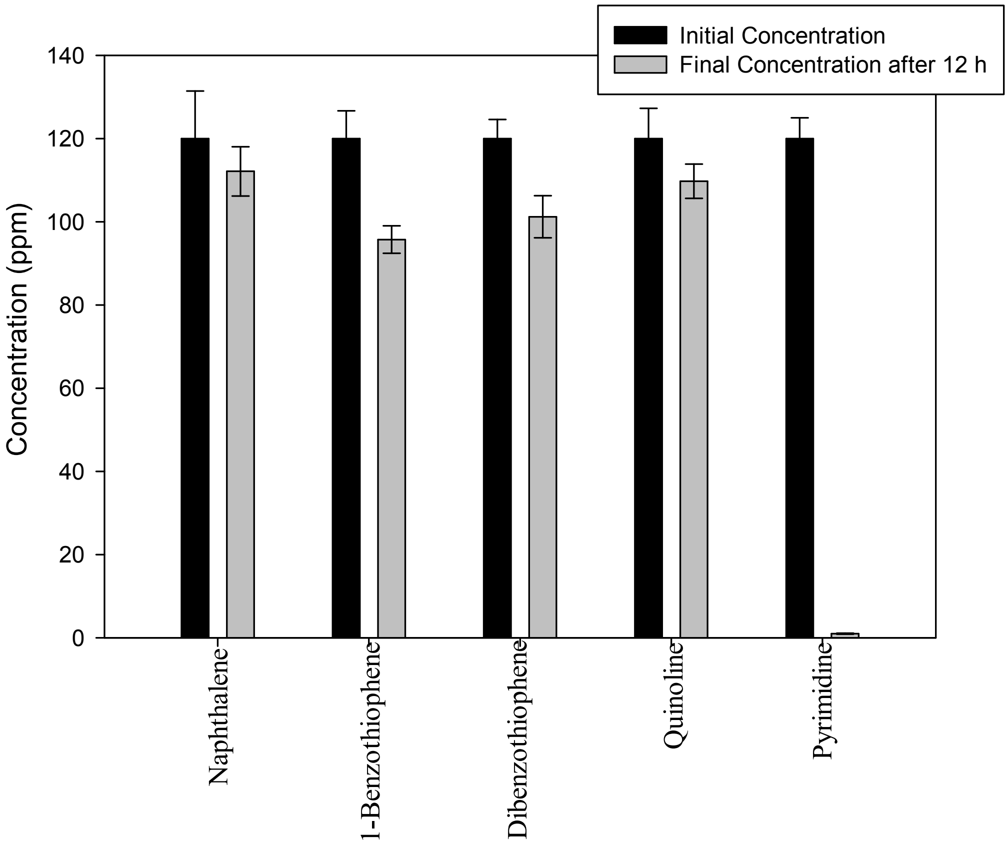

Figure S7. Adsorption selectivity of pyrimidine using pyrimidine-imprinted nanofibers.

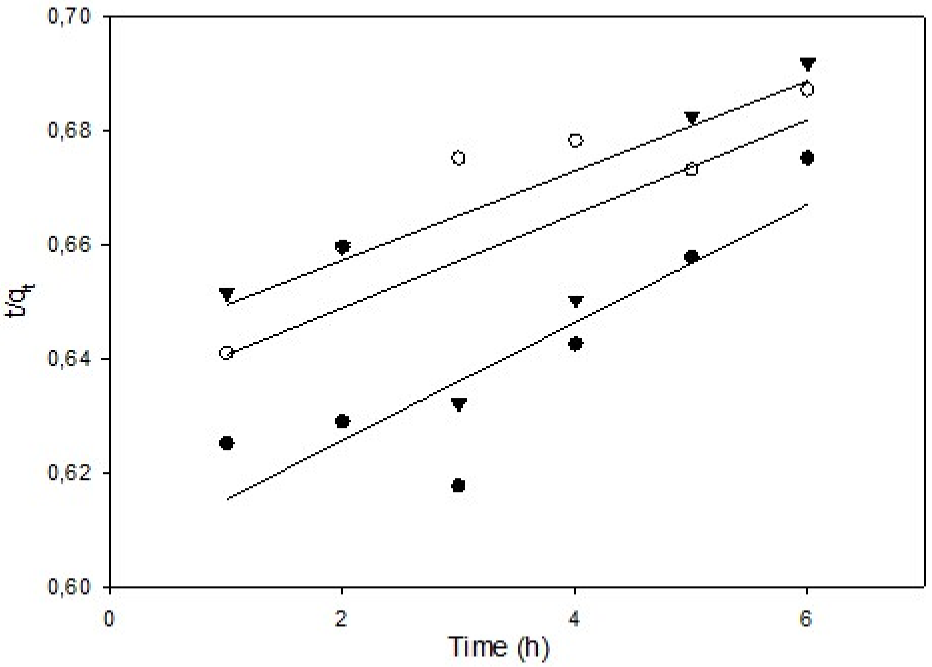

Figure S8: Pseudo-second-order plot of the nitrogen compounds (▼) pyrimidine, (○) carbazole and (●) quinoline.

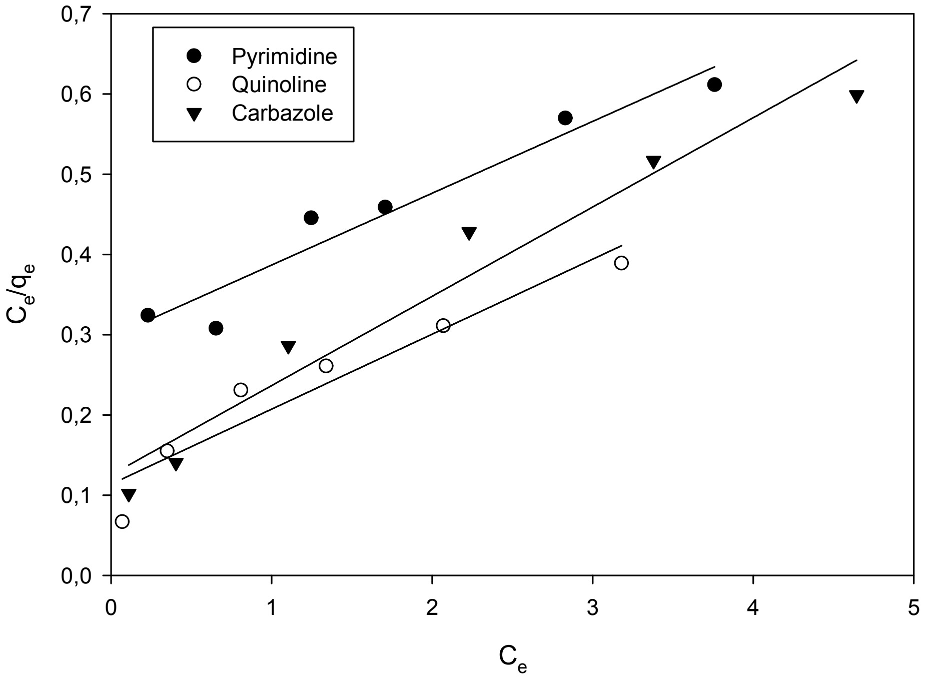

Figure S9. Langmuir plot for (A) carbazole, (B) quinoline and (C) pyrimidine.

Table S1: Imprinting factor nanofibers

Adsorbents	Qe (NIP)	Qe (MIP)	Imprinting Factor (k)
Pyrimidine	0.64	11.88	18.56
Quinoline	0.62	11.74	18.92
Carbazole	0.51	11.30	22.16

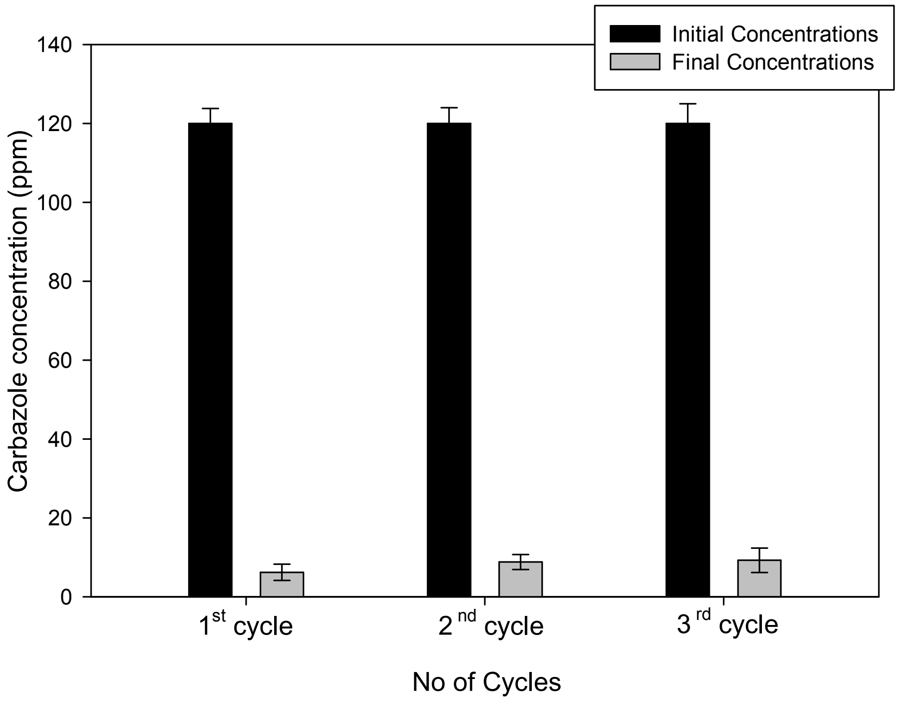

Figure S10. Reusability studies on the use of carbazole-imprinted nanofibers for the adsorption of carbazole.

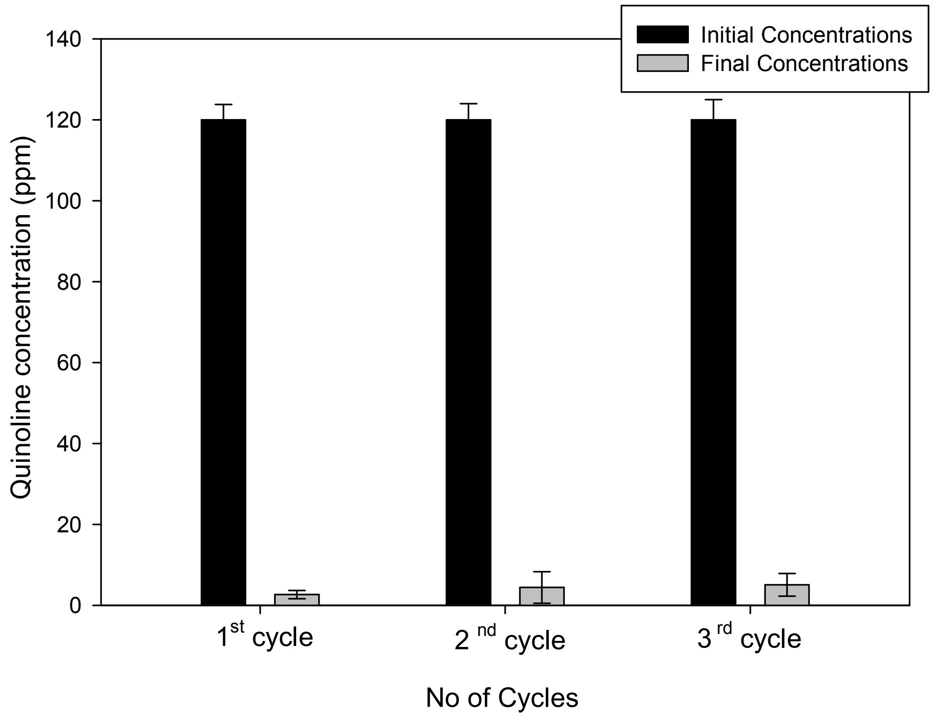

Figure S11. Reusability studies on the use of quinoline-imprinted nanofibers for the adsorption of quinoline.

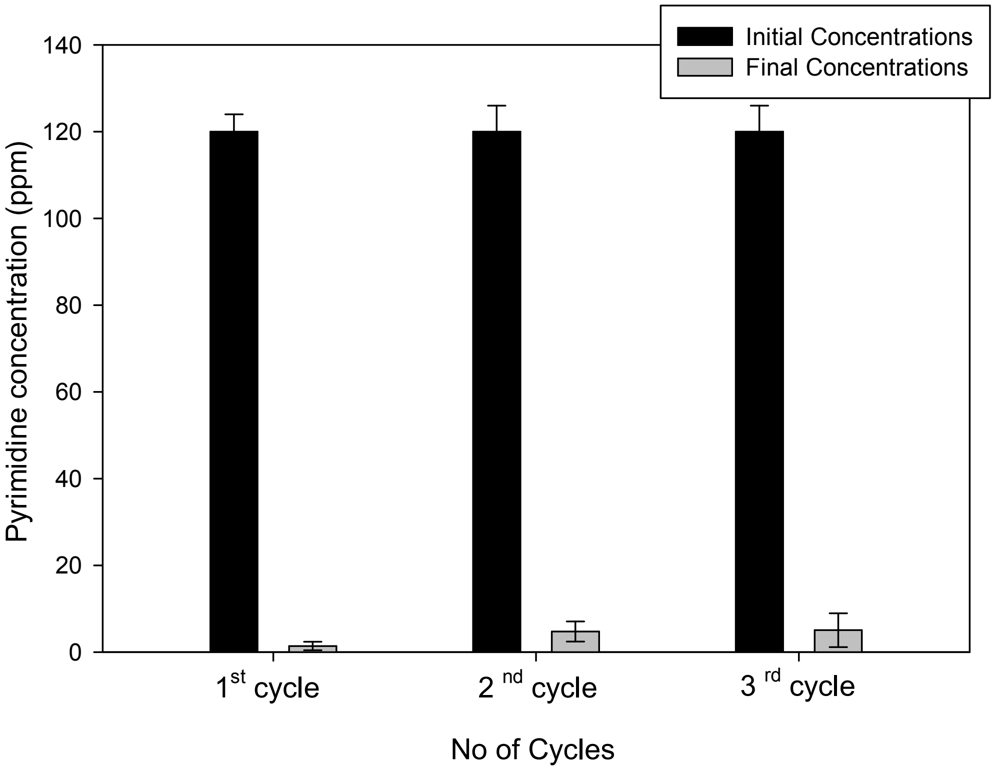

Figure S12. Reusability studies on the use of pyrimidine-imprinted nanofibers for the adsorption of pyrimidine.

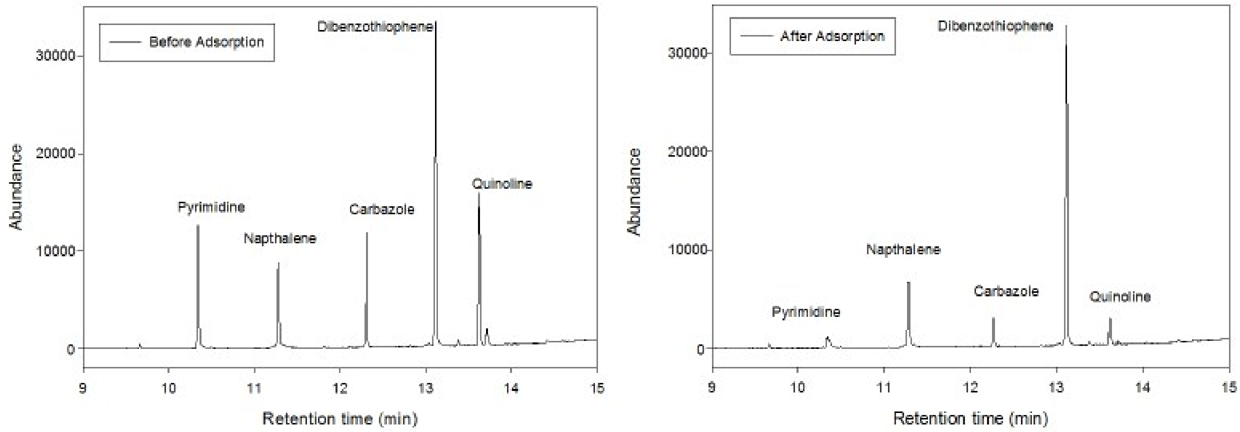

Figure S13. GC-FID chromatograms of model fuel before and after adsorption with imprinted PIMH nanofibers.

Table S2. Some electronic structure identifiers of the studied ligand and adducts

Compounds	E_H (a.u.)	E_L (a.u.)	Orbital Energy Gap. (E_G) (a.u.)	[a]Orbital Energy Gap. (E_G) (eV)
PIMH	-0.19759	0.01822	0.17937	4.88091
Carbazole (CAR)	-0.20870	-0.03452	0.17418	4.73698
Pyrimidine (PYM)	-0.25482	0.04252	0.21230	5.77698
Quinoline (QUN)	-0.23116	-0.05082	0.18034	4.90730
PIMH-CAR	-0.19955	-0.03297	0.16658	4.53288
PIMH-PYM	-0.19317	-0.05823	0.13494	3.67191
PIMH-QUN	-0.19198	-0.06496	0.12702	3.45639

[a]The energies of HOMO, LUMO gaps energies were converted from a.u. to eV using the conversion factor of 27.2114.

Section A

Synthesis of 2-(1H-imidazol-2-yl)-4-vinylphenol (PIMH)

1.1 Synthesis of the precursor 4-bromo-2-(1H-imidazol-2-yl) phenol

4-Bromo-2-hydroxybenzaldehyde (10 g, 0.05 mol) and glyoxal trimer dehydrate (10.5 g, 0.05 mol) were added in 200 mL glacial acetic acid and brought to reflux under nitrogen until dissolved then ammonium acetate (36 g, 0.05 mol) was added followed by continuous reflux for 4 hours. After cooling to room temperature, the solution was poured into 2 L water and filtered on celite. The dark brown filtrate was made basic by addition of aqueous ammonia; a yellow precipitate was formed and filtered dried. The product was dissolved in acetone and refluxed with activated carbon for an hour. This was filtered on celite and concentrated with rotavapor to give a purple product weighing 1.5 g. This was further purified on silica column with ethyl acetate and ethanol to elute (ratio 1:1). This was now concentrated using rotavap and the solid product weighed (scheme 1). Yield = 22 %. Melting point = 240-242°C. FT-IR (cm^{-1}, neat): 3368, 2414, 1586, 1530, 1494, 1463, 1280, 1252. ^{1}H NMR (400 MHz, DMSO-d_6) 12.44 (s, 1H), 8.09 (d 1H), 7.38 (d 2H), 7.07 (br-s, 2H), 6.92-6.90 (d, 1H). ^{13}C NMR (400 MHz, DMSO-d_6) 159.86, 155.49, 131.98, 126.62, 123.98, 119.90, 115.34, 110.62. *Anal. Calcd* (found) for $C_9H_7BrN_2O$ (%): C, 45.22 (44.95) H, 2.95 (3.203) N, 11.72 (10.71).

1.2 Synthesis of 2-(1-benzoyl-1H-imidazol-2-yl)-4-bromophenyl benzoate

4-Bromo-2-(1H-imidazol-2-yl) phenol (0.8 g, 0.003 mol) was dissolved in 30 mL THF and triethylamine (0.61 g, 0.006 mol) was added followed by benzoyl chloride (0.84 g, 0.006 mol). This was stirred overnight the solution was filtered and the filtrate concentrated to give a purple solid which was digested in diethyl ether and washed in the same solvent. A yield of 40% was observed (scheme 1). Yield = 15.13%. Melting point = 131-133°C. FT-IR (cm^{-1} neat): 1738, 1664, 1600, 1577, 1474, 1444, 1396, 1260. ^{1}H NMR (400 MHz, DMSO-d_6): 7.83 (m, 2H), 7.74 (M 1H), 7.52 (m 5H), 7.56 (m s. 1H), 7.42 (M 4H).^{13}C NMR (400 MHz, DMSO-d_6) 166.64, 163.57, 147.12, 134.11, 134.03, 133.07, 133.37, 130.75, 134.11, 130.34, 129.58, 129.17, 128.51, 127.88, 125.04, 121.97, 118.01. *Anal. Calcd* (found) for $C_{23}H_{15}BrN_2O_3$ (%) C, 61.76 (60.08) H, 3.38 (3.55) N. 6.26 (6.42).

1.3 *Synthesis of 2-(1-benzoyl-1H-imidazol-2-yl)-4-vinylphenyl benzoate*

2-(1-Benzoyl-1H-imidazol-2-yl)-4-bromophenyl benzoate (1 g, 2.24 mol) was dissolved in warm toluene. Palladium tetrakis(triphenylphosphine) (0.052 g, 0.045 mol) was added tributyl (vinyl) tin (0.65 mL, 2.24 mol) was added and a trace of 3,5-diterbutylcatechol was added. The mixture was heated to reflux under nitrogen for 5 h. After cooling, it was filtered and the filterate concentrated to give a yellow sticky liquid. This was dissolved in 40 mL acetonitrile and 20 mL hexane was added for washing the resulting solution. The lower of the two-immiscible liquid was run into a beaker it is yellow and contains the product. This was concentrated and gave a yellow solid to which diethyl ether was added which coagulated it into solid it was filtered. Yield: 85 % (scheme 1). Melting point = 122-124 °C. FT-IR (cm^{-1} neat): 1737, 1710, 1600, 1500, 1450, 1259. ^1H NMR (400 MHz, DMSO-d_6): 7.83 (m, 2H), 7.76 (s, 1H), 7.61-7.48 (m, 5H), 7.34-7.22 (m, 5H), 7.20 (M, 2H), 6.80 (m, 1H vinyl-H), 5.90 (d, 1H vinyl-H), 5.36 (d, 1H vinyl- H); ^{13}C NMR (400 MHz, DMSO-d_6): 166.78, 163.79, 147.26, 144.08, 133.94, 130.87, 129.54, 128.78, 128.46, 122.95, 121.65, 115.28. *Anal. Calcd.* (Found) (%) for $C_{25}H_{18}N_2O_3$ C, 76.13 (76.05) H, 4.60 (4.84), N, 7.10 (6.28).

1.4 *Synthesis of 2-(1H-imidazol-2-yl)-4-vinylphenol (PIMH)*

0.45 of 2-(1-benzoyl-1*H*-imidazol-2-yl)-4-vinylphenyl benzoate was dissolved in 10 mL of ethanol sodium ethoxide was prepared separately by adding 50 mg of sodium in dry ethanol (50 mg). The sodium ethoxide was then added to the product solution drop wise till the mixture becomes yellow it is concentrated and dissolved in 50 mL of 0.20 mol NaOH. The pH was adjusted to 10 by adding 0.1 mol HCl until the solution becomes cloudy the precipitate was filtered and centrifuged and then wash with ice cold methanol followed by cold diethyl ether giving a pure cream solid (scheme 1). Yield: 37.7% Melting point = 133-134°C FT- IR (cm^{-1}, neat) 3369, 2918, 1609, 1532, 1494, 1464, 1413, 1279, 1253. ^1H NMR (400 MHz, DMSO-d_6): 14.28 (s, 1H) 8.05 (s, 1H) 7.33 (d, 1H) 6.90 (d, 1H) 6.70-6.62 (m, 1H vinyl-H) 5.90-5.86 (d, 1H vinyl-H) 5.23-5.20 (d, 1H vinyl-H). ^{13}C NMR (400 MHz, DMSO-d_6): 155.50, 144.86, 131.98, 127.81, 126.63, 118.97, 115.35, 109.87. *Anal. Calcd* (found) for $C_{11}H_{10}N_2O$ (%): C, 70.97 (71.00); H, 5.40 (5.41); N, 15.02 (15.01). ^1H-NMR spectra of (A) 4-bromo-2-(1*H*-imidazol-2-yl)

phenol, (B) 2-(1-benzoyl-1*H*-imidazol-2-yl)-4-bromophenyl benzoate, (C) 2-(1-benzoyl-1*H*-imidazol-2-yl)-4-vinylphenyl benzoate and (D) 2-(1*H*-imidazol-2-yl)-4-vinyl phenol are all presented in Figure S1 of the supplementary section.

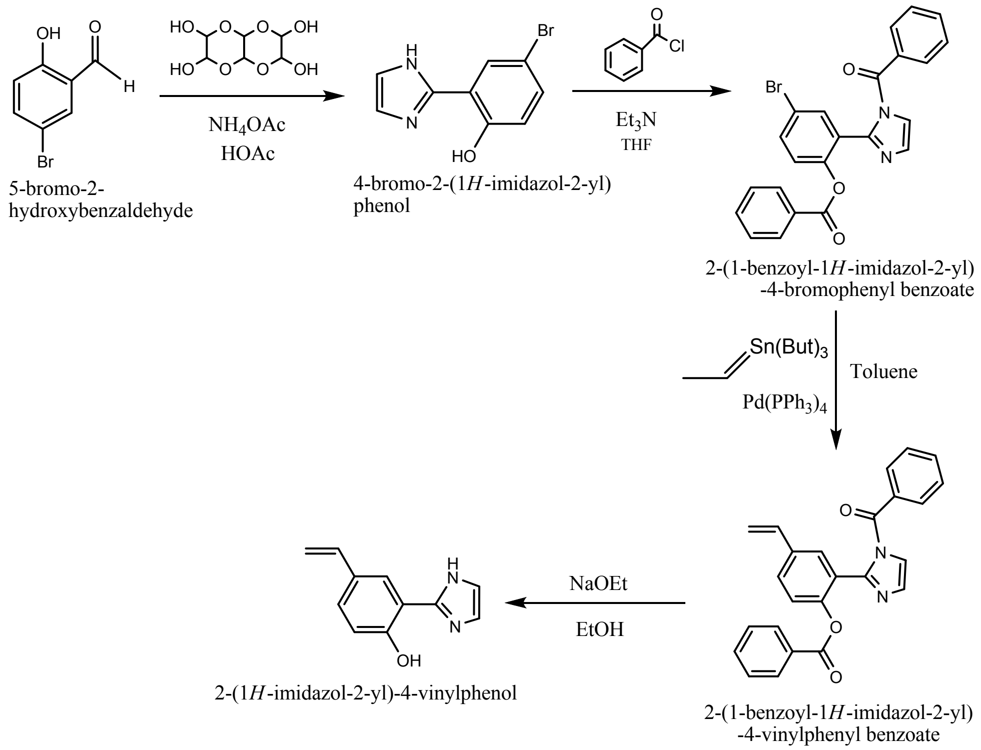

Scheme 1: The synthesis scheme for 2-(1*H*-imidazol-2-yl)-4-vinyl phenol

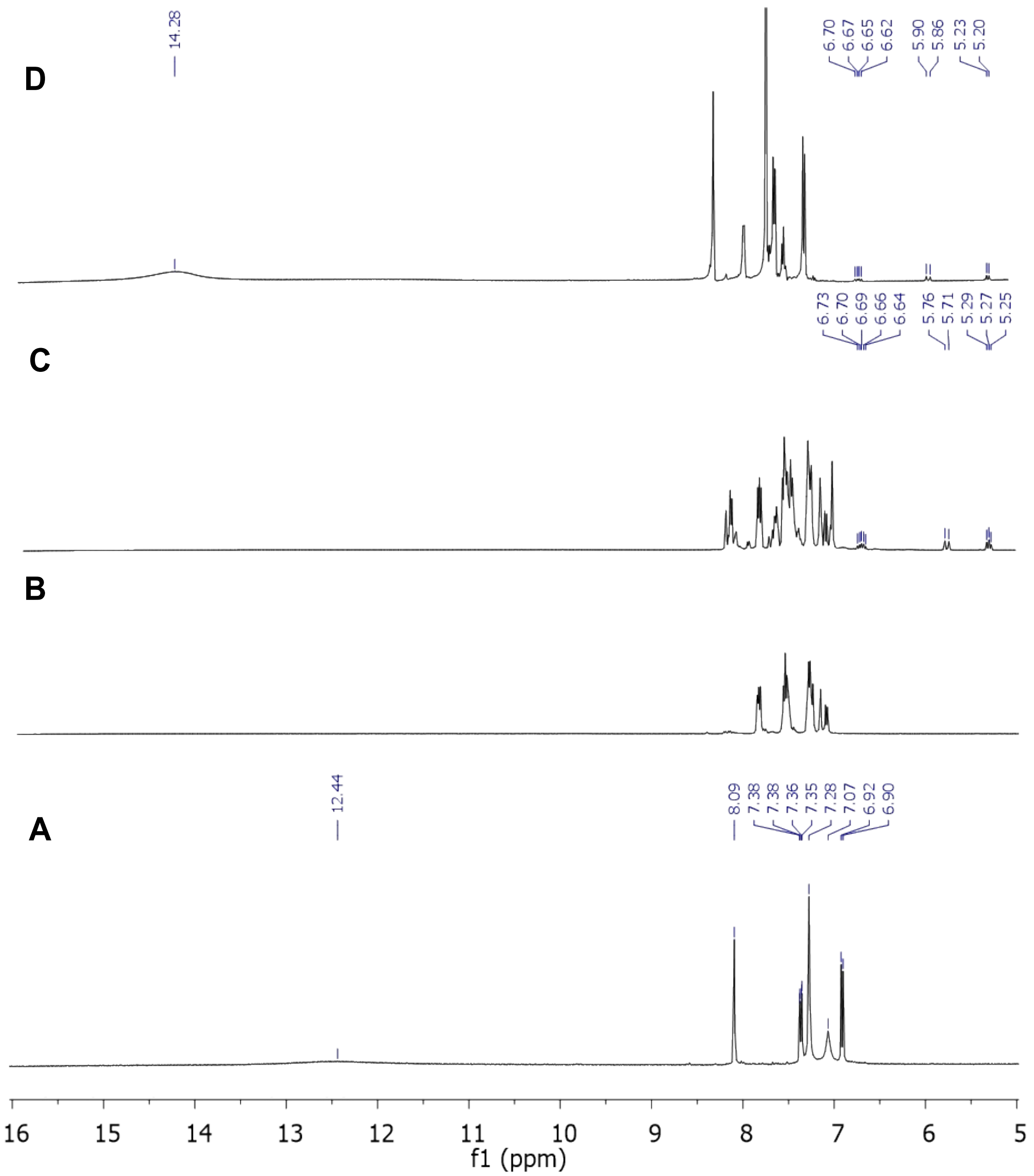

Figure 1: ^{1}H-NMR spectra of (A) 4-bromo-2-(1*H*-imidazol-2-yl) phenol, (B) 2-(1-benzoyl-1*H*-imidazol-2-yl)-4-bromophenyl benzoate, (C) 2-(1-benzoyl-1*H*-imidazol-2-yl)-4-vinylphenyl benzoate and (D) 2-(1*H*-imidazol-2-yl)-4-vinyl phenol.

Section B

2.1 Theoretical Studies

Molecular interactions between poly 2-(1*H*-imidazol-2-yl)-4-phenol (PIMH) and the various nitrogen compounds (quinoline, carbazole and pyrimidine) were modelled and discussed in the supplementary data section.

HOMO-LUMO gap: The HOMO−LUMO gap describes the stability and resistance of molecules, and it also predicts reactivity between species by providing the electrical transport properties as well as electron carrier and mobility in molecules. The HOMO-LUMO gap describes the degree of interaction strength between the various nitrogen compounds and 2-(1*H*-imidazol-2-yl)-4-phenol (PIMH), as lower values indicate higher strengths of interaction.

Interaction studies: The atomic level interaction of the various nitrogen compounds (quinoline, carbazole and pyrimidine) with poly 2-(1*H*-imidazol-2-yl)-4-phenol were predicted by molecular interaction studies using B3LYP functional with a basis set 6-311G++(d,p) using the Gaussian09 software (calculated at 298K) [1] under tight convergence criteria. The enthalpies of formation, Gibb's free energies and entropy of formation were calculated by using Eqn. 1 and 2, respectively.

$$\Delta\Delta H_{adduct} = \Delta H_{adduct} - (m\Delta H_{PIMH} + t\Delta H_{NCs}) \qquad (1)$$

where *m* and *t* are the stoichiometric amounts of PIMH and the individual nitrogen compounds (NCs) involved in adduct formation.

$$\Delta\Delta G_{adduct} = \Delta\Delta H_{adduct} - T\Delta\Delta S_{adduct} \qquad (2)$$

ΔΔG, T and ΔΔS are the Gibbs free energy for the adduct formation, temperature (298 K) and entropy for adduct formation at standard conditions (i.e. 1 molar concentration for solvents and 1 atm pressure) respectively. Results obtained from theoretical studies were compared to experimental-Isothermal Titration Calorimetry (ITC).

The interactions on a molecular level depend basically on the following interactions, and these are interaction between the HOMO and LUMO orbitals, π-π interaction, hydrogen bond interaction and van der Waals forces. A molecule with low HOMO energy indicates a better electron donor (high electronegativity), while a molecule with high LUMO energy indicate better electron acceptor (high electron affinity).

Hardness (ŋ), softness (σ), electronegativity (χ) and chemical potential (μ) were calculated using the following equations;

$$ŋ = \frac{I - A}{2} \qquad (3)$$

$$\sigma = \frac{1}{ŋ} \qquad (4)$$

$$\chi = \frac{I + A}{2} \qquad (5)$$

$$\mu = -\chi \qquad (6)$$

According to the Koopmans theorem, the ionization energies and the electron affinities of the chemical species are calculated using the following equations;

$I = -E_{HOMO}$

$A = -E_{LUMO}$

Finally, LUMO-HOMO energy gap (ΔE) was calculated using the following formula; $\Delta E = E_{LUMO} - E_{HOMO}$.

2.2 Isothermal Titration Calorimetry (ITC)

Isothermal titration calorimetry (ITC) which is based on the detection of the heat released or consumed upon titration of an analyte with (sub)microliter aliquots of a titrant, was employed to follow the degree of analyte-molecule interactions in solution [2]. This technique has found widespread application across many different research fields, particularly in the quantification of host-guest and supramolecular assemblies [3, 4], metal ion [5] as well as biomolecular interactions [6-12].

For this experiment, high-sensitivity ITC experiments were carried out at 25°C. N-compounds-PIMH titration was performed on a modular titration nanocalorimeter TAM III with an injection volume of 10 μL, a time spacing of 10 mins between injections and a stirrer speed of 40 rpm. The reference cell was left empty.

Results

Thermodynamic parameters such as enthalpy (ΔΔH) entropy (ΔΔS) and free energies (ΔΔG) resulting from adduct formation are presented in Table 2. A decrease in free energy of the system ($\Delta G_b < 0$)

confirmed that the adsorption process was spontaneous. A decrease in the randomness of the interacting molecules give rise to negative entropies ($\Delta S_b < 0$). Negative enthalpy (ΔH_b) value resulting from interaction molecules contributed to an exothermic process. Theoretical studies agreed with experimental studies (ITC) as interaction between 2-(1*H*-imidazol-2-yl)-4-phenol and nitrogen compounds {quinoline and pyrimidine} were favourable, spontaneous and exothermic in nature. While the interaction between 2-(1*H*-imidazol-2-yl)-4-phenol and carbazole is endothermic and non-spontaneous.

Table 2. DFT molecular modelling thermodynamic data ($\Delta\Delta H$, $\Delta\Delta G$ and $\Delta\Delta S$) on the formation of adduct between nitrogen compounds {quinoline, carbazole and pyrimidine} and 2-(1*H*-imidazol-2-yl)-4-phenol.

	ΔΔG ($kcal.mol^{-1}$)	ΔΔH ($kcal.mol^{-1}$)	ΔΔS ($kcal.mol^{-1}$)
Quinoline	-6.327	-15.999	-0.032
Carbazole	0.529	5.729	-0.021
Pyrimidine	-6.375	-14.373	-0.026

2.4 Thermodynamic studies

2.4.1 Stoichiometry of the interaction-Isothermal Titration Calorimetry (ITC)

The binding isotherms were carried out using the isothermal titration calorimetry (ITC). The same titration conditions were employed (i.e. concentrations, injection volumes, etc.) for nitrogen compounds {quinoline, carbazole and pyrimidine} with 2-(1*H*-imidazol-2-yl)-4-vinyl phenol. The ITC interaction titrations between 2-(1*H*-imidazol-2-yl)-4-vinyl phenol (PIMH) with (A) quinoline, (B) pyrimidine and (C) carbazole are presented in figure 4. The peaks displayed the progress of binding of the N-compounds to 2-(1*H*-imidazol-2-yl)-4-vinyl phenol with time. The amount of heat released as interaction progresses decrease, hence confirming a decrease in adsorption capacity (Fig. 4).

From the obtained thermodynamic parameters, negative free energies were observed for all interactions (quinoline-PIMH, pyrimidine-PIMH and carbazole-PIMH) (Table 3). With quinoline-PIMH and pyrimidine-PIMH interactions proceeding spontaneously than carbazole/PIMH. During the adsorption

process, the randomness of the interacting molecules decreases, hence giving rise to negative entropies (-VE ΔS_b). Pyrimidine-PIMH presented a lower degree of randomness as compared to quinoline-PIMH and carbazole-PIMH interactions. The positive value of ΔS obtained for carbazole-PIMH suggests increased randomness at the solid/solution interface with some structural changes in the adsorbate and adsorbent. Based on the obtained enthalpy (ΔH_b) values obtained, quinoline-PIMH and pyrimidine-PIMH offer better interaction as compared carbazole-PIMH (Table 3). The stoichiometry of interaction (n), binding constant (K), free energy (ΔG_b), enthalpy (ΔH_b) and entropy (ΔS_b) is presented in Table 3.

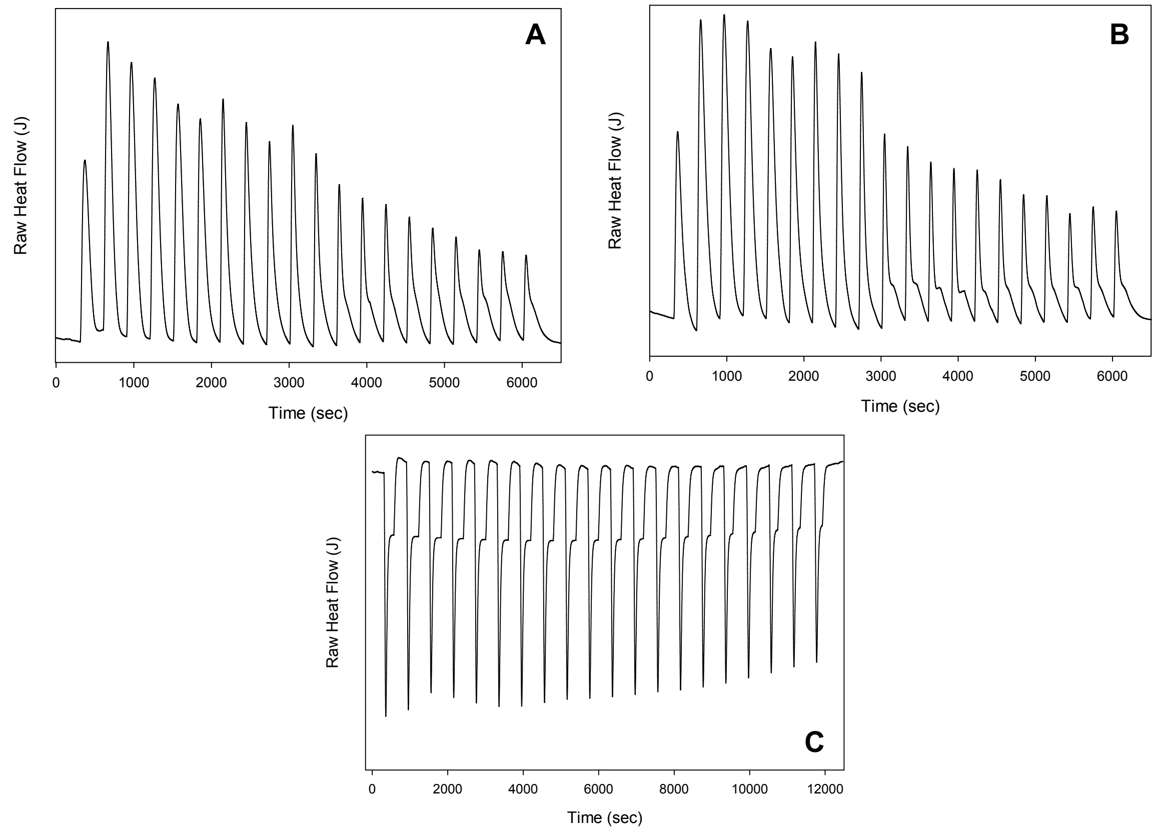

Figure 4: ITC titration involving 2-(1*H*-imidazol-2-yl)-4-vinyl phenol (PIMH) with (A) quinoline, (B) pyrimidine and (C) carbazole. 100 mM (quinoline/pyrimidine/carbazole) was titrated into 20 mM 2-(1*H*-imidazol-2-yl)-4-vinyl phenol (PIMH).

Table 3 Thermodynamic properties obtained from Isothermal titration calorimetry (ITC).

	Free energy (ΔG_b)	Stoichiometry of the interaction (n)	Enthalpy (ΔH_b) (kJ/mol)	Entropy (ΔS_b) (kJ/mol)	k	Temperature (K)

	(kJ/mol)					
PIMH-Quinoline	-28.8	$9.87\times10^{-1} \pm 4.6\times10^{-2}$	-18.0±4.8	-10.3	1.1×10^{2}	298
PIMH-Pyrimidine	-30.3	$9.14\times10^{-1} \pm 5\times10^{-2}$	-28.1±3.6	-35.3	2.0×10^{2}	298
PIMH-Carbazole	-4.3	$1.13 \pm 1\times10^{-2}$	3.3 ±0.7	10.9	1.8×10^{1}	298

References

1. M. J. Frisch, G. W. Trucks, H. B. Schlegel, G. E. Scuseria, M. A. Robb, J. R. Cheeseman, G. Scalmani, V. Barone, B. Mennucci, G. A. Petersson, H. Nakatsuji, M. Caricato, X. Li, H. P. Hratchian, A. F. Izmaylov, J. Bloino, G. Zheng, J. L. Sonnenberg, M. Hada, M. Ehara, K. Toyota, R. Fukuda, J. Hasegawa, M. Ishida, T. Nakajima, Y. Honda, O. Kitao, H. Nakai, T. Vreven, J. A. Montgomery, Jr., J. E. Peralta, F. Ogliaro, M. Bearpark, J. J. Heyd, E. Brothers, K. N. Kudin, V. N. Staroverov, R. Kobayashi, J. Normand, K. Raghavachari, A. Rendell, J. C. Burant, S. S. Iyengar, J. Tomasi, M. Cossi, N. Rega, J. M. Millam, M. Klene, J. E. Knox, J. B. Cross, V. Bakken, C. Adamo, J. Jaramillo, R. Gomperts, R. E. Stratmann, O. Yazyev, A. J. Austin, R. Cammi, C. Pomelli, J. W. Ochterski, R. L. Martin, K. Morokuma, V. G. Zakrzewski, G. A. Voth, P. Salvador, J. J. Dannenberg, S. Dapprich, A. D. Daniels, Ö. Farkas, J. B. Foresman, J. V. Ortiz, J. Cioslowski, and D. J. Fox, Gaussian, Inc., Wallingford CT, 2009.

2. G. Krainer, J. Broecker, C. Vargas, J. Fanghänel and S. Keller, Quantifying High-Affinity Binding of Hydrophobic Ligands by Isothermal Titration Calorimetry Anal. Chem. 2012, 84, 10715-10722.

3. A. Cooper, M. A. Nutley, P. Camilleri, Microcalorimetry of Chiral Surfactant−Cyclodextrin Interactions. Anal. Chem., 1998, 70, 5024-5028.

4. Adeniyi S. Ogunlaja, E. Hosten, R. Betz, Zenixole R. Tshentu, Selective removal of isoquinoline and quinoline from simulated fuel using 1,1'-binaphthyl-2,2'-diol (BINOL): Crystal structure and evaluation of the adduct electronic properties. RSC Adv., 2016, 6, 39024 - 39038.

5. C. Schönbeck, R. Holm and P. Westh, Higher Order Inclusion Complexes and Secondary Interactions Studied by Global Analysis of Calorimetric Titrations. Anal. Chem., 2012, 84, 2305-2312.

6. S. Leavitt, E. Freire, Direct measurement of protein binding energetics by isothermal titration calorimetry. Curr. Opin. Struct. Biol., 2001, 11, 560-566.

7. L. N. Wafer, W. W. Streicher, S. A. McCullum, G. I. Makhatadze, Thermodynamic and Kinetic Analysis of Peptides Derived from CapZ, NDR, p53, HDM2, and HDM4 Binding to Human S100B. Biochemistry 2012, 51, 7189-7201.

8. O. Khakshoor, S. E. Wheeler, K. N. Houk and E. T. Kool, Measurement and Theory of Hydrogen Bonding Contribution to Isosteric DNA Base Pairs. J. Am. Chem. Soc. 2012, 134, 3154-3163.

9. X. Wang, E. Matei, A. M. Gronenborn, O. Ramström, M. Yan, Direct Measurement of Glyconanoparticles and Lectin Interactions by Isothermal Titration Calorimetry. Anal. Chem., 2012, 84, 4248-4252.

10. S. Keller, H. Heerklotz, A. Blume, Monitoring Lipid Membrane Translocation of Sodium Dodecyl Sulfate by Isothermal Titration Calorimetry. J. Am. Chem. Soc., 2006, 128, 1279-1286.

11. H. Heerklotz, A. D. Tsamaloukas, Keller, S. Monitoring detergent-mediated solubilization and reconstitution of lipid membranes by isothermal titration calorimetry. Nat. Protoc. 2009, 4, 686-697.

12. M. M. Pierce, C. S. Raman, B. T. Nall, Isothermal Titration Calorimetry of Protein–Protein Interactions, Methods 1999, 19, 213-221.
